# Proven anti-virulence therapies in combating methicillin- and vancomycin-resistant *Staphylococcus aureus* infections

**DOI:** 10.3389/fcimb.2024.1403219

**Published:** 2024-08-26

**Authors:** Walid Bakeer, Marwa Gaafar, Ahmed O. El-Gendy, Mohamed. A. El Badry, Mona G. Khalil, Abdallah Tageldein Mansour, Nada K. Alharbi, Heba M. R. M. Selim, Mahmoud M. Bendary

**Affiliations:** ^1^ Department of Microbiology and Immunology, Faculty of Pharmacy, Beni-Suef University, Beni-Suef, Egypt; ^2^ Quality Control Specialist at Egyptian Drug Authority (EDA), Cairo, Egypt; ^3^ Department of Botany and Microbiology, Faculty of Sciences, Al- Azhar University, Cairo, Egypt; ^4^ Department of Pharmacology and Toxicology, Faculty of Pharmacy, Modern University for Technology and Information, Cairo, Egypt; ^5^ Department of Fish and Animal Production and Aquaculture, College of Agriculture and Food Sciences, King Faisal University, Al-Ahsa, Saudi Arabia; ^6^ Department of Fish and Animal Production, Faculty of Agriculture (Saba Basha), Alexandria University, Alexandria, Egypt; ^7^ Department of Biology, College of Science, Princess Nourah bint Abdulrahman University, Riyadh, Saudi Arabia; ^8^ Department of Pharmaceutical Sciences, College of Pharmacy, AlMaarefa University, Riyadh, Saudi Arabia; ^9^ Department of Microbiology and Immunology, Faculty of Pharmacy, Port Said University, Port Said, Egypt

**Keywords:** anti-virulence, histopathological, molecular docking, coumarin, simvastatin, ibuprofen

## Abstract

**Introduction:**

Despite years of efforts to develop new antibiotics for eradicating multidrug-resistant (MDR) and multi-virulent Methicillin-Resistant *Staphylococcus aureus* (MRSA) and Vancomycin-Resistant *Staphylococcus aureus* (VRSA) infections, treatment failures and poor prognoses in most cases have been common. Therefore, there is an urgent need for new therapeutic approaches targeting virulence arrays. Our aim is to discover new anti-virulence therapies targeting MRSA and VRSA virulence arrays.

**Methodology:**

We employed phenotypic, molecular docking, and genetic studies to screen for anti-virulence activities among selected promising compounds: Coumarin, Simvastatin, and Ibuprofen.

**Results:**

We found that nearly all detected MRSA and VRSA strains exhibited MDR and multi-virulent profiles. The molecular docking results aligned with the phenotypic and genetic assessments of virulence production. Biofilm and hemolysin productions were inhibited, and all virulence genes were downregulated upon treatment with sub-minimum inhibitory concentration (sub-MIC) of these promising compounds. Ibuprofen was the most active compound, exhibiting the highest inhibition and downregulation of virulence gene products. Moreover, *in vivo* and histopathological studies confirmed these results. Interestingly, we observed a significant decrease in wound area and improvements in re-epithelialization and tissue organization in the Ibuprofen and antimicrobial treated group compared with the group treated with antimicrobial alone. These findings support the idea that a combination of Ibuprofen and antimicrobial drugs may offer a promising new therapy for MRSA and VRSA infections.

**Conclusion:**

We hope that our findings can be implemented in clinical practice to assist physicians in making the most suitable treatment decisions.

## Introduction

A key biological property of *Staphylococcus aureus* is its capacity to persist in various environments and on intact epithelia without being detected by the host. This silent presence allows *S. aureus* to spread easily and become a common part of the human microbiota (Taylor and Unakal, 2023). On the other hand, it is responsible for several diseases and a broad spectrum of potentially life-threatening infections, ranging from superficial skin abscesses to necrotizing tissue and pulmonary lesions, if it enters the bloodstream or internal tissues (Luzzago et al., 2014; Abd El-Hamid et al., 2022). Therefore, the relationship between *S. aureus* colonization, pathogenicity, and human diseases has been extensively studied (Hamdan-Partida et al., 2022). Furthermore, *S. aureus* is one of the most common human pathogens causing both community-acquired and nosocomial infections. It can produce different virulence factors that help in its adaptation and survival in various tissues and environmental conditions (Balasubramanian et al., 2017). Thus, recent clinical research has focused on the relationship between virulence arrays, pathogenicity, bacterial evolution, and host factors (Cheung et al., 2021). Several *S. aureus* virulence factors, including staphylokinase, leukocidin, invasive proteases, hemolysin, hyaluronidase, lipase, and nuclease, play important roles in colonization and pathogenicity, facilitating the initiation of the disease process, immune evasion, and host tissue destruction (Wieckowska-Szakiel et al., 2007).

Alarmingly, several life-threatening diseases have been associated with the multi-virulent strains of this pathogen. These strains create several public health crises, especially if they show resistance to available antimicrobial drugs. *S. aureus* can easily acquire resistance against all classes of antibiotics through targeted gene mutations or by horizontal transfer of resistance genes. The most notable example of these strains is methicillin-resistant *S. aureus* (MRSA), which is characterized by the presence of the *mecA* gene encoding penicillin-binding protein 2a. Originally a major cause of nosocomial infections, MRSA has now also become prevalent in community settings (Peacock and Paterson, 2015; Abd El-Hamid et al., 2023). Glycopeptides, particularly vancomycin, have been considered the most effective drugs against severe MRSA infections, as most MRSA strains are vancomycin-sensitive *S. aureus* (VSSA). However, there has been an alarming emergence of vancomycin-resistant *S. aureus* (VRSA), which may be attributed to the widespread use of vancomycin for the treatment of infections caused by MRSA (Cong et al., 2019; Rubinstein and Keynan, 2014).

In Egypt, the prevalence of MRSA has increased with significant variation across different regions and studies. A comprehensive meta-analysis of 64 studies revealed an overall MRSA prevalence of 63% among clinical isolates (Azzam et al., 2023). Recent data have indicated that the MRSA prevalence among clinical isolates ranges widely in this country, with rates between 50% and 82% in major cities such as Cairo and Alexandria and a lower rate of approximately 24% in Minia (Abouelfetouh, 2017). A study conducted in this area between 2005 and 2013 showed a significant rise in VRSA cases from 4.2% to 25.8% among hospital-associated infections (Saber et al., 2022). Another recent study has reported VRSA prevalence rates of 27.6% in dromedary camel isolates and 54.5% in human isolates (Al-Amery et al., 2019). These findings highlight a growing concern for antimicrobial resistance among the MRSA and VRSA isolates in the region. Therefore, the multidrug-resistant MRSA and VRSA strains have been implicated in serious problems and health crises throughout the world. This crisis has been compounded by the emergence of multi-virulent phenotypes.

In recent years, there has been an increase in resistance to available antimicrobial drugs, while antimicrobial development has lagged. Thus, the development and discovery of new drugs are urgently needed to overcome antimicrobial resistance, which has undermined the effectiveness of conventional antibiotics (Ghaly et al., 2020). In order to manage microbial infections, alternative anti-virulence therapies targeting virulence factors instead of microbial growth have been approved and have entered clinical trials by several health organizations worldwide (Dickey et al., 2017). It has been proven that virulence factors are involved in pathogenesis, while anti-virulence agents inhibit the production of disease-causing virulence factors, but are neither bactericidal nor bacteriostatic. Unlike antibiotics, which face problems related to the evolution and spread of resistance, the horizontal transfer of virulence genes cannot provide protection against anti-virulence therapies as they are correlated to single or very closely related species. Anti-virulence agents cannot be used as growth promoters in veterinary fields, in contrast to antibiotics, which is considered one of the major mechanisms responsible for antibiotic resistance. Furthermore, anti-virulence therapies have no effect on the normal microbiota. Thus, the superinfection issues associated with the use of antibiotics are not involved during treatment with anti-virulence therapies (Dickey et al., 2017). These new strategies may assist in suppressing the production of key MRSA and VRSA toxins. In addition, they might be useful alone or in combination with other antibiotics to manage MRSA and VRSA infections and overcome the currently unmet problems related to biofilm-producing pathogens. Herein, we attempted to address the antimicrobial resistance crisis through the evaluation of the anti-virulence activities of some natural (coumarin) and synthetic (simvastatin and ibuprofen) compounds using different *in vitro*, *in vivo*, and molecular docking techniques.

## Materials and methods

### Characterization of MRSA and VRSA strains

During a 3-month period from January 2022 to April 2022, a total of 250 clinical specimens were collected from both inpatients and outpatients admitted to a general hospital in Cairo, Egypt. These specimens were recovered from different clinical sources, including blood, urine, pus, and wound swabs. All clinical specimens were collected aseptically following standard methodology according to instructions and guidelines (Cheesbrough, 2005). All the tested specimens were inoculated and enriched in both trypticase soy agar (TSA) and blood agar (BA) and then incubated at 37°C for 24 h (Hudzicki, 2016). The recovered *S. aureus* isolates were identified phenotypically based on standard procedures for the identification of bacterial pathogens according to the provided instructions and guidelines (Koneman et al., 2006; Cheesbrough, 2017). In addition, biochemical identification was conducted using API 20S. MRSA strains were identified using cefoxitin antibiotic disks (30 μg) on Mueller–Hinton agar plates through the Kirby–Bauer disc diffusion method, evaluated in accordance with the Clinical and Laboratory Standards Institute guidelines (CLSI, 2022). The minimum inhibitory concentration (MIC) breakpoints of oxacillin were also determined using the broth microdilution method in triplicate. Isolates were classified as MRSA if they exhibited a zone of inhibition ≤21 mm around the cefoxitin disk and had an MIC ≥4 µg/mL. In addition, the agar dilution assay was employed in triplicate to determine the MIC for the identification of VRSA strains according to CLSI (2022). In brief, Mueller–Hinton agar plates were prepared with vancomycin concentrations ranging from 0.5 to 64 µg/mL. Subsequently, 1–2 µL of the standardized bacterial suspensions (0.5 McFarland standard) was added to the surface of the agar plates, typically with 24–36 spots per plate. The inoculated plates were then incubated in an inverted position at 35°C for 18–24 h. The MIC denotes the minimum vancomycin concentration necessary to entirely halt visible growth of *S. aureus*. As per the CLSI guidelines (2022), isolates are categorized as VRSA if their MIC is ≥16 µg/mL. Additional confirmation tests to verify the MRSA and VRSA strains were performed in triplicate using the Biomerieux VITEK®2, an automated Identification/Antibiotic Susceptibility Testing (ID/AST) system. For more confirmation, the E-TEST procedure for the detection of VRSA strains was performed in trypticase as follows: firstly, a standardized inoculum of the bacterial strain was prepared by adjusting its turbidity to match the 0.5 McFarland standard. This inoculum was then evenly spread over a Mueller–Hinton agar plate using a sterile swab. After allowing the plate to dry for a few minutes, an E-TEST strip, which contains a gradient of vancomycin concentrations, was carefully placed on the agar surface. The plate was incubated at 35–37°C for 24 h. Following incubation, the MIC was determined by identifying the point at which the elliptical zone of inhibition intersects with the E-TEST strip. The MIC values were then interpreted to identify the resistant strains (Maharjan et al., 2021).

### Screening of multiple drug-resistant MRSA and VRSA strains

The antimicrobial susceptibility testing of all identified isolates was conducted *in vitro* on 20 different commonly prescribed antimicrobial agents, covering various categories, therapeutic actions, and mechanisms of action. These antimicrobials included cefoxitin (FOX; 30 μg), benzylpenicillin (P; 10 U), amoxicillin/clavulanic acid (AMC; 20/10 μg), gentamicin (CN; 10 μg), clindamycin (DA; 2 μg), nitrofurantoin (NFT; 300 μg), linezolid (LZD; 30 μg), vancomycin (VA; 30 μg), teicoplanin (TEC; 30 μg), tetracycline (TET; 30 μg), erythromycin (E; 15 μg), doxycycline (DO; 30 μg), ciprofloxacin (CIP; 5 μg), levofloxacin (LEV; 5 μg), chloramphenicol (C; 30 μg), fusidic acid (FA; 10 μg), rifampicin (RD; 5 μg), cotrimoxazole (SXT; 25 μg), and pristinamycin (quinupristin/dalfopristin) (QD; 15 μg). This test was performed using the Kirby–Bauer disc diffusion method according to the criteria and guidelines of CLSI (2022). Isolates that showed resistance to at least one drug in three or more antimicrobial categories were identified as multidrug-resistant (MDR) (Magiorakos et al., 2012).

### Phenotypic detection of some virulence factors of the identified MRSA and VRSA isolates

#### Quantitative determination of biofilm formation using the microtiter plate method

The microtiter plate (MTP) method was performed according to the standard procedures and instructions provided by Lade et al. (2019). In brief, biofilm production was assessed using the MTP method with crystal violet staining. Bacterial cultures were initially grown in 96-well plates, followed by washing to eliminate non-adherent cells after incubation. The residual biofilm was then stained with crystal violet and subsequently washed to remove surplus dye. To quantify the biofilm, the stained biofilm was dissolved using acetone, and its optical density was measured using a spectrophotometer. The experiment was conducted in triplicate using sterile trypticase soy broth (TSB) as a negative control, which was used as the cutoff point to quantify the biofilm formation ability [optical density cutoff (ODc) = average OD of the negative control + 3× standard deviation of the negative control]. The isolates were classified according to Stepanović et al. (2007) into the following categories: strong biofilm producer (4 × ODc < OD), moderate biofilm producer (2 × ODc < OD ≤ 4 × ODc), weak biofilm producer (ODc < OD ≤ 2 × ODc), and no biofilm production (OD ≤ ODc).

#### Qualitative and quantitative detection of hemolysin production

A blood plate assay was used for the determination of both qualitative and quantitative hemolytic activity, which was performed three times to detect the average of measurements (Yigit and Aktas, 2009; Nouraei et al., 2020). The hemolysin-producing strain was characterized by the production of a clear zone. Hemolytic activity was determined in millimeters by measuring the diameter of the inoculate divided by the diameter of the clear zone around the inoculate. The precipitation zone was measured and expressed as a *Pz* value from 1 to 4 as follows: *Pz* 1 (negative) for no clearance, *Pz* 1+ (0.9–1) for mild, *Pz* 2+ for moderate (0.89–0.80), *Pz* 3+ (0.79–0.70) for strong, and *Pz* 4+ (≥0.69) for very strong hemolysin activity (Divyakolu et al., 2019; Almwafy et al., 2020).

#### Genotypic detection of some virulence factors of selected MRSA and VRSA strains

The MDR, hemolysin, and biofilm-producing MRSA and VRSA isolates were selected from the examined samples for further genotypic analysis. This molecular study examined 13 virulence genes, including five enterotoxin genes (*sea*, *seb*, *sec*, *sed*, and *see*), which were detected using multiplex PCR. In addition, three hemolysin genes (*hla*, *hlb*, and *hlg*), two intracellular adhesion toxin genes (*icaA* and *icaD*), one toxic shock syndrome toxin gene (*tst*), one exfoliative toxin B gene (*etb*), and one leukocidin gene (*lukED*) were investigated. All of these genes were detected with uniplex PCR. Negative controls (DNA extracted from the *Escherichia coli* reference strain ATCC 25922) and the positive control (DNA extracted from standard *S. aureus*, ATCC 33591) were used as controls for each PCR run. The standard strains were kindly provided by the Animal Production Research Institute in Dokki, Giza, Egypt. Electrophoresis of the amplified PCR products on an agarose gel (1.5%) stained with ethidium bromide (Sigma-Aldrich, St. Louis, MO, USA) allowed for their visualization under UV light. The primer sequences, annealing temperatures, and amplicon sizes are shown in **Supplementary Table S1**.

### Assessment of anti-virulence compounds (coumarin, simvastatin, and ibuprofen) using molecular docking studies

Molecular docking analysis was used to evaluate the affinity scores of coumarin, simvastatin, and ibuprofen to specific virulence gene product targets *in vitro* and to predict any anti-virulence activity against virulence proteins. The virulence proteins included in the Protein Data Bank (PDB) were leukocidin, penicillin-binding protein (PBP), toxic shock syndrome toxin (TSST), enterotoxins, exfoliative toxin B, intracellular adhesive toxin A, and hemolysin. The sum of all interactions between the tested compounds and their target proteins was approximated with a docking score. The predicted top-ranking poses with the best scores were detected using the AutoDock Vina, GOLD, and MOE-Dock programs. GOLD and LeDock were able to identify the correct ligand binding poses. Both Glide (XP) and GOLD predicted the poses consistently with 90% accuracy (Wang et al., 2018).

#### Evaluation of the antimicrobial and anti-virulence activities of the selected compounds

The antimicrobial and anti-virulence effects of coumarin, simvastatin, and ibuprofen were evaluated against the three most highly multi-virulent MRSA/VRSA strains, as well as the standard *S. aureus* (ATCC 33591).

#### Determination of the MICs of the investigated compounds

For this evaluation, 100 μL of Mueller–Hinton broth medium was added to each well of a MTP. Subsequently, twofold serial dilutions of each drug were prepared in the respective wells of each row. Following this, a standardized suspension (100 μL) of the MRSA isolates (0.5 McFarland standard) was added to each well. During this analysis, two controls were utilized: a positive control (culture medium of the susceptible strain with the antimicrobial agent) and a negative control (culture medium without the antimicrobial agent). The final volume in each well of the MTP was 200 μL. The plates were sealed, placed in plastic bags, and incubated at 35°C for a total of 24 h. Absorbance was determined with an enzyme-linked immunosorbent assay (ELISA) reader at a wavelength equal to 630 nm (Flentie et al., 2019). The MIC was determined as the lowest concentration of the antimicrobial agent that entirely halted visible bacterial growth.

#### Evaluation of the anti-biofilm activities of coumarin, simvastatin, and ibuprofen

The biofilm-producing multi-virulent/MDR isolates were selected to examine the anti-biofilm activities of the compounds under investigation. Sub-MIC (half MIC value) concentrations of coumarin, simvastatin, and ibuprofen were employed to evaluate their anti-biofilm activities using the MTP method (Lade et al., 2019). The test was repeated three times, with both positive and negative controls included in each repetition.

#### Evaluation of the anti-hemolytic activities of coumarin, simvastatin, and ibuprofen

Blood samples (5 mL) from physiologically normal volunteers were centrifuged at 5,000 rpm for 10 min to separate plasma and erythrocytes. The supernatant was discarded, and the erythrocytes were collected and purified by washing three times using phosphate-buffered saline. Aliquots of 50 µL of erythrocytes were treated with 25 µL of sub-MIC (MIC = 0.5) of the tested compounds in a Falcon tube and incubated at room temperature for 60 min. The positive control, which was treated with Triton-X100 resulting in 100% hemolysis, and the negative control, an untreated tube, were both employed. The OD at 540 nm was measured using a UV spectrophotometer to reflect the degree of hemolysis (Yigit and Aktas, 2009; Afsar et al., 2016). The percentage of anti-hemolysis was calculated using the following equation: % inhibition = 100 × (1 - OD sample) / OD of negative control.

#### Molecular detection of the anti-virulence activities of the investigated compounds using the real-time PCR method

RNA was extracted from the top three highly multi-virulent MRSA/VRSA strains along with the standard *S. aureus* (ATCC 33591) following the instructions provided in the RNeasy Mini Kit. The amplification conditions were set on a thermocycler, which included reverse transcription at 50°C for 30 min, initial denaturation at 94°C for 15 min, followed by 40 cycles of secondary denaturation at 94°C for 30 s, annealing at 57°C for 40 s, and extension at 72°C for 40 s. Subsequently, a dissociation curve was generated with denaturation at 94°C for 1 min, annealing at 57°C for 1 min, and a final denaturation at 94°C for 1 min. The amplification curves and the cycle threshold (Ct) values were determined using the Stratagene MX3005P software. To assess variations in the gene expression among the RNA samples, the Ct value of each sample was compared with that of the control group using the “ΔΔCT” method outlined by Yuan et al. (2006). Specifically, ΔΔCt was calculated as ΔCt reference − ΔCt target, where ΔCt (target) = Ct (control) − Ct (treatment) and ΔCt (reference) = Ct (control) − Ct (treatment). The dissociation curves were compared between different samples to eliminate false-positive results.

### *In vivo* and histopathology studies

#### Animals

Adult male Wistar rats weighing 200–250 g were obtained from the animal facility at the National Research Centre (Giza, Egypt). They were maintained on a standard laboratory diet and provided unlimited access to tap water. The experimental animals were housed in a temperature-controlled room at 22–25°C with a 12-h light/dark cycle. Care for all animals adhered to ethical standards, and the study protocols followed institutional guidelines for the use of laboratory animals. The research was approved by the Ethics Committee for Animal Experimentation at the Faculty of Pharmacy, Port Said University (approval no. REC.PHARM.PSU.22–7), and complied with the Guide for the Care and Use of Laboratory Animals published by the US National Institutes of Health (NIH publication no. 85–23, revised 2011).

### Experimental design

Four groups, each consisting of 15 rats, were classified as follows: group 1 (G1), which served as the negative control group (non-infected, untreated control group); group 2 (G2), the positive control group consisting of infected rats left untreated; and group 3 (G3) and group 4 (G4), which comprised challenged rats with the highest multi-virulent VRSA strain. G3 was treated topically with vancomycin, while G4 received a combination of vancomycin and the most potent anti-virulence compound. All rats underwent anesthesia using ketamine and xylazine before their back hair was shaved. Subsequently, a full-thickness wound was created using a 20-mm diameter biopsy puncher. Immediately after wound induction, 5 μL [1 × 10^5^ colony forming unit per milliliter (CFU/ml)] of VRSA was applied to the wounds of rats in G2, G3, and G4. Following 24 h of bacterial inoculation, G3 and G4 commenced receiving topical application of vancomycin and the combination of vancomycin/the most potent anti-virulence compound on the wounds, respectively. The change in the wound healing area was monitored over a span of 3 weeks, with wound measurements taken using a caliper on days 3, 5, 10, 14, and 21 post-initial wounding. In addition, the total aerobic microbial count was assessed in all four groups on days 3, 5, 10, 14, and 21. Finally, the rats were euthanized and the skin tissue samples from the wound area dissected out for histopathological examination (Nagappa and Cheriyan, 2001).

### Determination of *in vivo* antibacterial activity and wound healing ability of vancomycin/the most potent anti-virulence compound combination

An animal model experiment on wound healing was conducted to assess both the antibacterial efficacy and the healing potential of the combination of vancomycin/the most potent anti-virulence compound on infected skin wounds (Nagappa and Cheriyan, 2001; Van de Vyver et al., 2021). The percentage of wound healing area was calculated using the following equation: Wound area % = (*W_t_*/*W*_i_) × 100, where, *W_t_* is the wound area at a certain time point and *W*_i_ is the initial wound healing area.

### Determination of the total aerobic microbial count

The total aerobic microbial count was assessed in the four groups on days 3, 10, and 14 using Dehydrated Plate Count Agar. The aseptic technique was employed, and the initial dilution was prepared from the sample to a 99-mL sterile saline blank, resulting in a 1/100 or 10^−2^ dilution. The CFU per milliliter or gram of the sample was determined by dividing the number of colonies by the dilution factor and then multiplying by the volume of specimen added to the liquefied agar. The CFU was calculated using the formula: Number of colonies (CFUs) = # of bacteria/mL dilution × amount plated.

### Histopathological and healing score assessment

At the end of days 3, 5, 10, 14, and 21 post-initial wounding, the mice were euthanized and the skin tissue samples from the wound area were excised and placed in neutral buffered formalin (10%) for fixation. The samples were then processed in xylenes and alcohols before embedding in paraffin wax. Sections of 5 μm were sliced and subjected to hematoxylin and eosin (H&E) staining for examination under light microscopy (Bancroft and Gamble, 2008). An Olympus CX43 light microscope (Olympus, Tokyo, Japan) equipped with a ToupTek camera (ToupTek, Hangzhou, China) was utilized to examine the tissue slides and to capture images. The histologic wound healing score (Abdellatif et al., 2021) was used to evaluate granulation tissue formation, re-epithelialization, inflammation, and angiogenesis at the wound site. The scoring criteria for re-epithelialization were as follows: 4 = Complete epidermal remodeling; 3 = Moderate epithelial proliferation; 2 = Incomplete epidermal organization; 1 = Poor epidermal organization; and 0 = Absence of epithelial proliferation. For the granulation tissue and collagen matrix organization, the scores were: 4 = Complete organized tissue; 3 = Thick granulation layer and well-formed collagen; 2 = Moderate remodeling; 1 = Thin immature and inflammatory tissue; and 0 = Immature granulation and inflammatory tissue. The degree of inflammation was assessed as follows: 4 = 13–15 inflammatory cells per histological field; 3 = 10–13 inflammatory cells per histological field; 2 = 7–10 inflammatory cells per histological field; 1 = 4–7 inflammatory cells per histological field; and 0 = 1–4 inflammatory cells per histological field. Angiogenesis was evaluated based on the number of vessels per site and associated features, with scores ranging from 4 = more than seven vessels per site arranged vertically toward the epithelial surface; 3 = five to six vessels per site, slight edema, and congestion; 2 = three to four vessels per site, moderate edema, and congestion; 1 = one to two vessels per site with edema, hemorrhage, and congestion; and 0 = absence of angiogenesis and presence of congestion, hemorrhage, and edema.

### Statistical analysis

Qualitative data were presented as percentages and frequencies, while numerical data were expressed as mean, standard error, standard deviation, or median, with appropriate ranges. Analysis of variance (ANOVA) was conducted using a two-factor model without replication. Both categorical and numerical data analyses were performed using SPSS software (version 20; SPSS, Chicago, IL, USA). A *p*-value <0.05 was considered statistically significant. In addition, GraphPad Prism version 6 (GraphPad Software Inc., San Diego, CA, USA) and the R packages corrplot, heatmaply, ggpubr, and hmisc were employed for the construction of all heat maps in this study. Moreover, correlation coefficients (*r* values) were utilized to evaluate the associations between biofilm production and clinical samples, as well as the different strain types. Positive correlations were indicated by *r* values greater than 0, while negative correlations were observed when the *r* values were less than 0 (Elmanakhly et al., 2024).

## Results

### Prevalence of MRSA and VRSA isolates

Among the 250 clinical specimens, 62 isolates of *S. aureus* (24.8%) were confirmed using standard bacteriological methods and API 20S. These isolates were confirmed based on the genetic amplification of the specific 16S rRNA gene. Interestingly, 43.5% (27/62) of these isolates exhibited resistance to cefoxitin and oxacillin antibiotics and thus were classified as MRSA strains. On the other hand, 11.3% (7/62) of the detected *S. aureus* isolates were identified as VRSA strains based on broth microdilution and the Biomerieux VITEK®2 automated ID/AST system, with MIC breakpoints ≥16 µg/mL. Furthermore, the MIC values determined by the E-TEST were in agreement with those obtained using both the agar dilution method and the Biomerieux VITEK®2 automated ID/AST system, confirming seven VRSA strains with MIC values ≥16 µg/mL. It is worth noting that all VRSA strains also displayed resistance to cefoxitin and oxacillin antibiotics (seven VRSA out of 27 MRSA isolates). The majority of the MRSA and VRSA isolates were found in pus specimens; however, the urine and blood samples showed the lowest rates of infection with MRSA and VRSA isolates, respectively. Both MRSA and VRSA isolates were more prevalent among female samples compared with male samples.

### Antimicrobial susceptibility profiles of the MRSA and VRSA isolates

**Figure 1:**
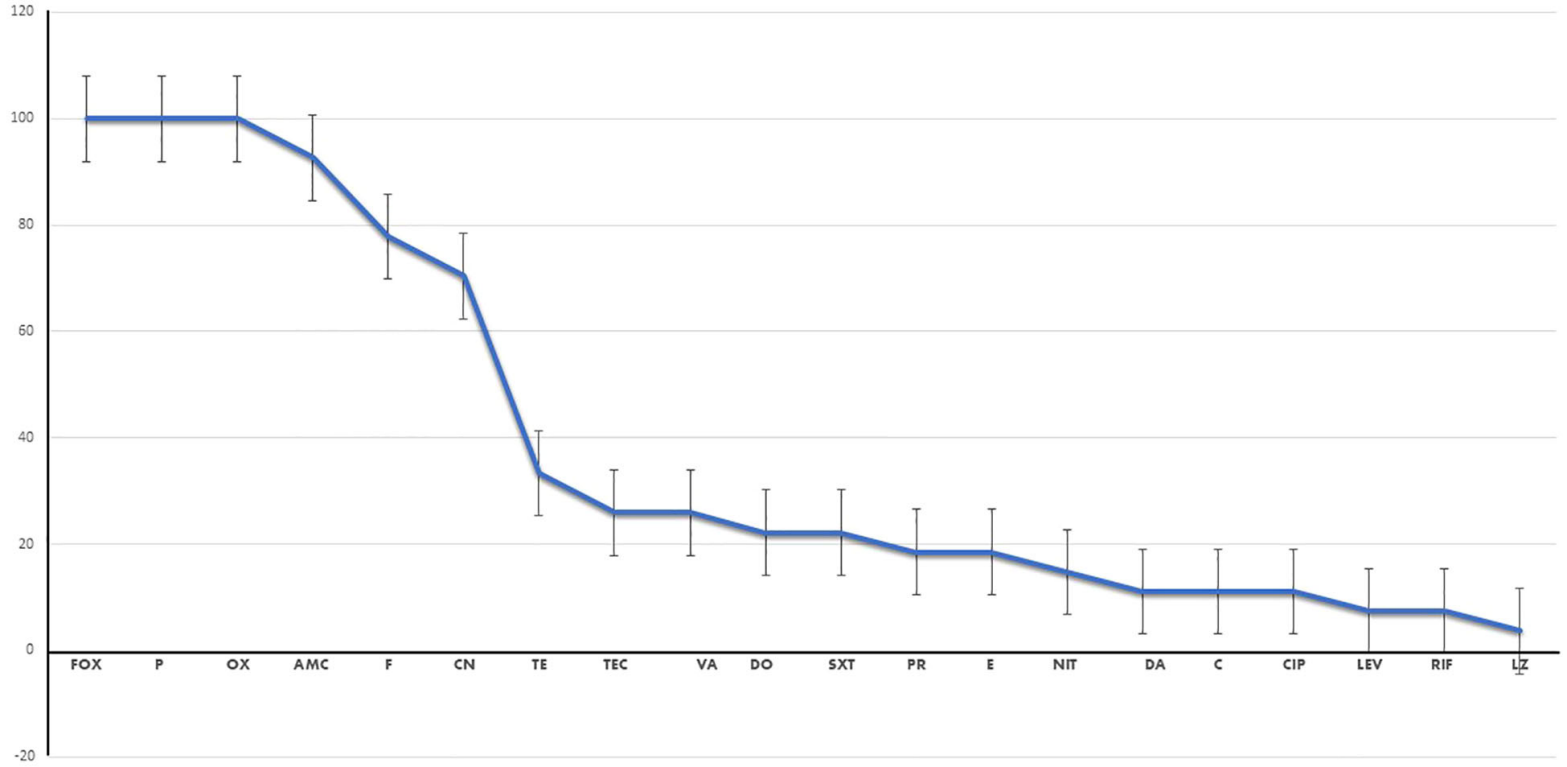
Percentage of resistance to each antimicrobial drug of the investigated isolates. *Fox*, cefoxitin; *P*, benzylpenicillin; *OX*, oxacillin; *AMC*, amoxicillin/clavulinic acid; *CN*, gentamicin; *E*, erythromycin; *DA*, clindamycin; *NFT*, nitrofurantoin; *LEZ*, linezolid; *VA*, vancomycin; *TEC*, teicoplanin; *TE*, tetracycline; *DC*, doxycycline; *CIP*, ciprofloxacin; *LEV*, levofloxacin; *C*, chloramphenicol; *FA*, fusidic acid; *RF*, rifampicin; *TMP/SXT*, cotrimoxazole; *QD*, pristinamycin (quinupristin/dalfopristin).

Regrettably, all 27 MRSA isolates exhibited MDR patterns, indicating resistance to three or more antimicrobial classes, as depicted in **Figure 1**. These isolates demonstrated complete resistance to β-lactam antibiotics such as cefoxitin, penicillin G, and oxacillin, as well as β-lactams combined with β-lactamase inhibitors such as amoxicillin/clavulanic acid. As anticipated, the antimicrobial resistance patterns observed in the VRSA isolates were particularly alarming when compared with those of the VSSA isolates. Both VSSA and VRSA isolates exhibited high resistance rates to fusidic acid, gentamicin, and tetracycline; however, they demonstrated relatively lower resistance rates to nitrofurantoin and rifampicin. Fortunately, all of the VSSA strains and over 50% of the VRSA strains displayed sensitivity to pristinamycin (quinupristin/dalfopristin) and linezolid, as illustrated in **Supplementary Figure S1**.

### Phenotypic identification of the hemolysin- and biofilm-producing MRSA and VRSA strains

All 27 MRSA isolates under investigation exhibited clear hemolytic zones on BA plates, indicating their capacity as hemolysin producers. Approximately 48.1% (13 out of 27) of the MRSA isolates were confirmed as biofilm producers using the MTP method (**Supplementary Figures S1**, **S2**). Among these 13 biofilm-producing MRSA isolates, six were VRSA isolates and seven were VSSA isolates (**Supplementary Figure S1**). Statistical analysis revealed that the source of the isolates could serve as a predictor of MRSA biofilm formation, as there was a significant association between the isolate source and biofilm formation. A strong positive correlation was observed between biofilm production and both wound swab and urine samples, with a slightly weaker correlation for the pus samples (*r* > 0). However, a negative association was noted with the blood samples (*r* < 0). Similarly, biofilm production was consistently found among the VRSA strains (*r* > 0), while non-biofilm production was prevalent among the VSSA strains (*r* < 0), as shown in **Supplementary Figure S3**.

### Molecular detection of virulence genes

**Figure 2:**
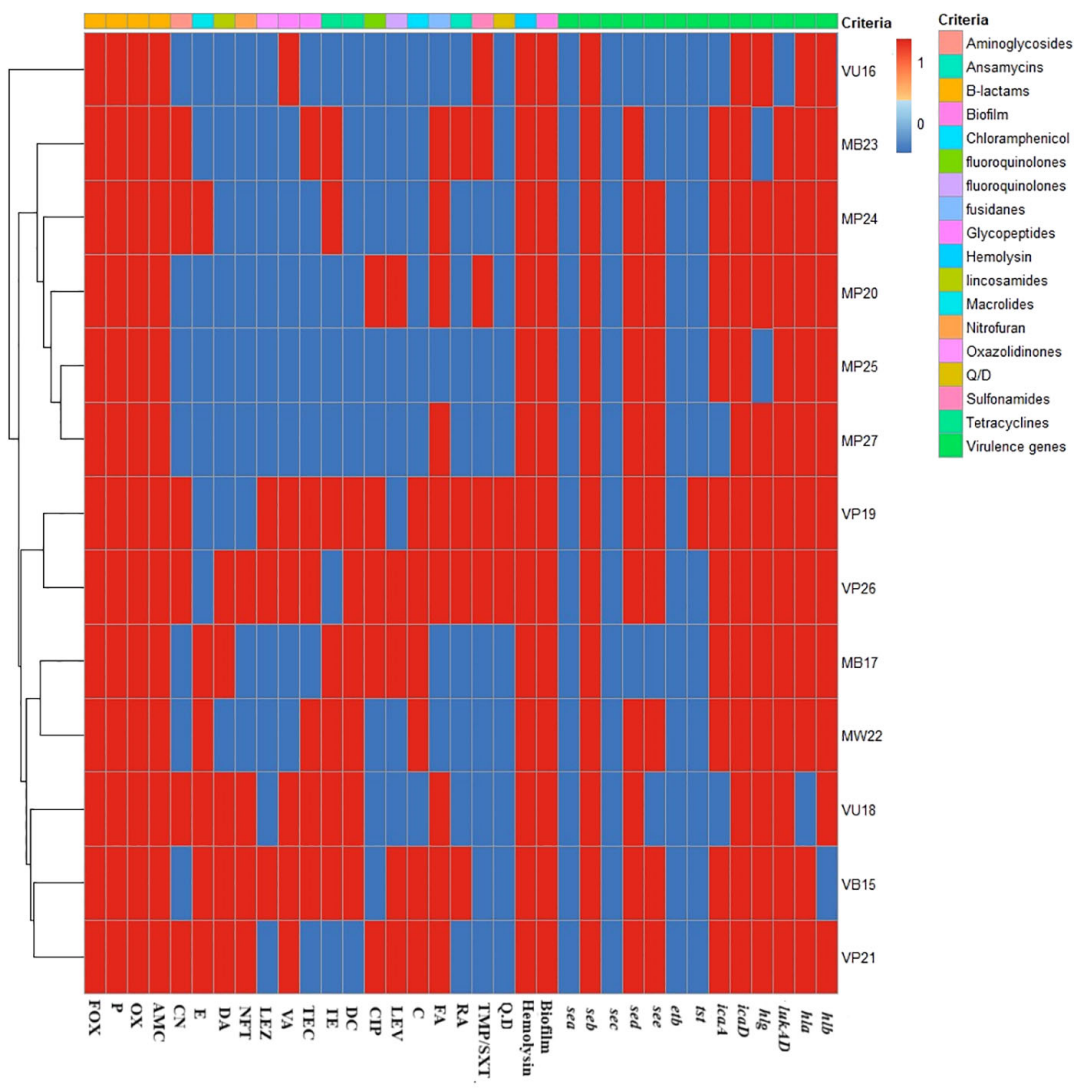
Heat map displaying the antimicrobial resistance and virulence gene profiles. For the isolate code, *M* and *V* represent the methicillin-resistant *Staphylococcus aureus* (MRSA) and vancomycin-resistant *S. aureus* (VRSA) strains, respectively. However, the *second letter* refer to the type of samples from which the isolate was recovered (i.e., *P*, pus; *B*, blood; *U*, urine; *W*, wound swab), and the *numerical values* refer to the order of recovery. The *red* and *blue colors* refer to the presence or the absence of resistance to each antimicrobial, biofilm, and hemolysin production, respectively. *Fox*, cefoxitin; *P*, benzylpenicillin; *OX*, oxacillin; *AMC*, amoxicillin/clavulinic acid; *CN*, gentamicin; *E*, erythromycin; *DA*, clindamycin; *NFT*, nitrofurantoin; *LEZ*, linezolid; *VA*, vancomycin; *TEC*, teicoplanin; *TE*, tetracycline; *DC*, doxycycline; *CIP*, ciprofloxacin; *LEV*, levofloxacin; *C*, chloramphenicol; *FA*, fusidic acid; *RF*, rifampicin; *TMP/SXT*, cotrimoxazole; *QD*, pristinamycin (quinupristin/dalfopristin). *sea*, *seb*, *sec*, *sed*, and *see* are the staphylococcal enterotoxins A, B, C, D, E genes; *hla*, *hlb*, and *hlg* are the hemolysin A, B, and G genes; *icaA* and *icaD* are the intracellular adhesive toxins A and D; *tst* is the toxic shock syndrome toxin gene; *etb* is the exfoliative toxin B gene; and *LukED* is the leukocidin gene.

Molecular analysis was conducted on 13 isolates, consisting of seven MRSA and six VRSA isolates, which were MDR, hemolysin, and biofilm producers, as illustrated in **Figure 2**. The virulence profiles of the examined isolates were striking, with all of them exhibiting multi-virulent profiles and harboring more than six virulence genes, with the exception of one isolate. The molecular analysis documented the presence of both the *icaD* and *seb* virulence genes in all the examined isolates (100%). Furthermore, all of the investigated isolates carried at least one of the hemolysin genes (i.e., *hla*, *hlb*, and *hlg*), with approximately 69.2% of these isolates possessing all three hemolysin genes; however, 92.3% of the tested isolates harbored *hla* or *hlb*, whereas *hlg* was detected in 84.6% of the investigated isolates.

Significantly, the *lukED*, *icaA*, and *tst* genes were detected in 92.3%, 76.9%, and 7.7% of the isolates in this study, respectively. The *seb*, *sed*, and *see* genes, on the other hand, were prevalent among the examined isolates at rates of 100%, 84.6%, and 69.2%, respectively. However, none of the examined isolates carried the *sec* and *sea* genes (**Figure 2**).

### Molecular docking analysis of coumarin, simvastatin, and ibuprofen to predict their affinity scores for the MRSA virulence genes

**Figure 3:**
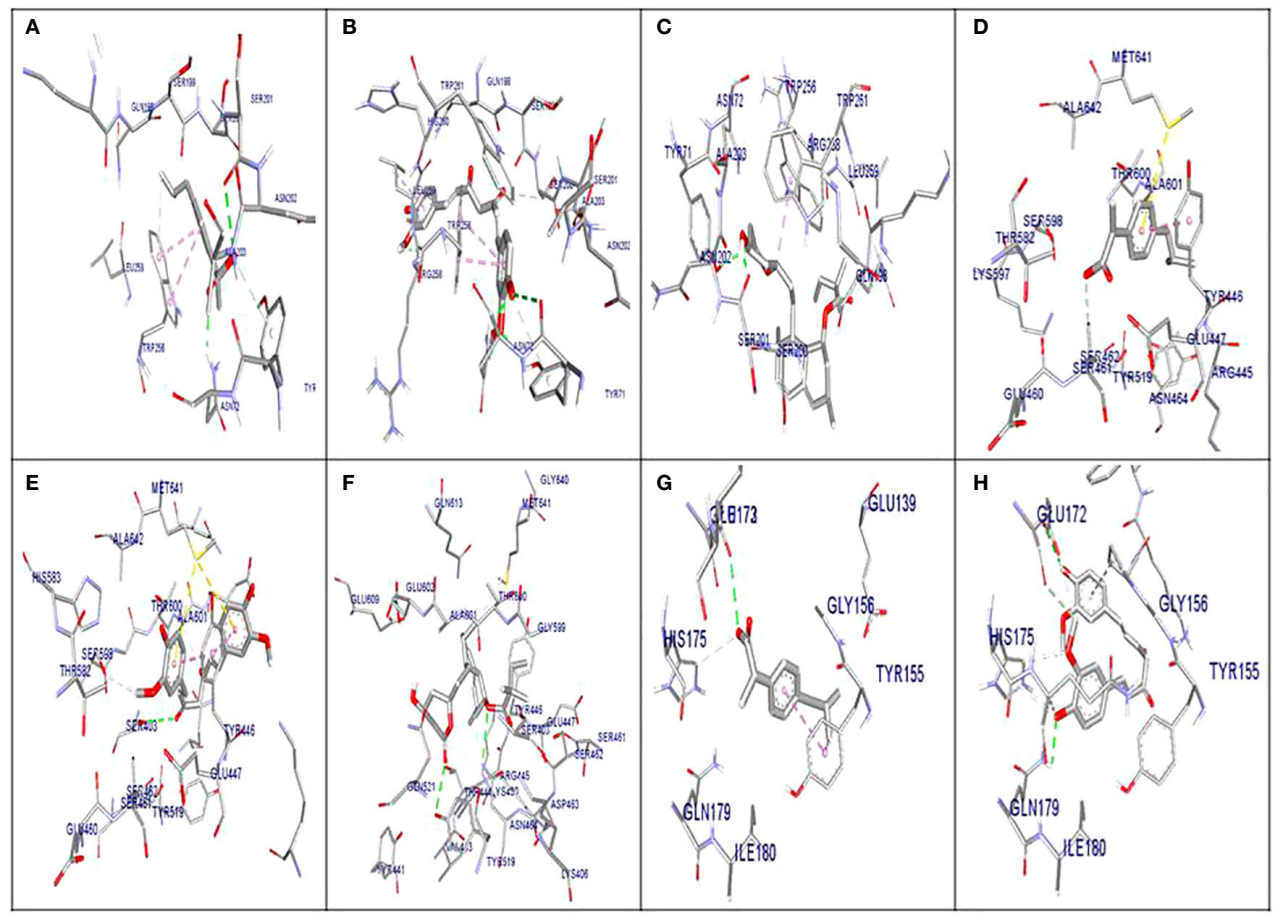
Molecular docking of the investigated compounds with the *lukED*, *pbp*, and *tst* virulence genes. **(A–C)** Three-dimensional visual representations of the drugs coumarin **(A)**, ibuprofen **(B)**, and simvastatin **(C)** showing bond formation and position in the active site of *lukED*. **(D–F)** Three-dimensional visual representations of the drugs coumarin **(D)**, ibuprofen **(E)**, and simvastatin **(F)** showing bond formation and position in the active site of *pbp*. **(G, H)** Three-dimensional visual representations of coumarin **(G)** and ibuprofen **(H)** showing bond formation and position in the active site of *tst*.

**Figure 4:**
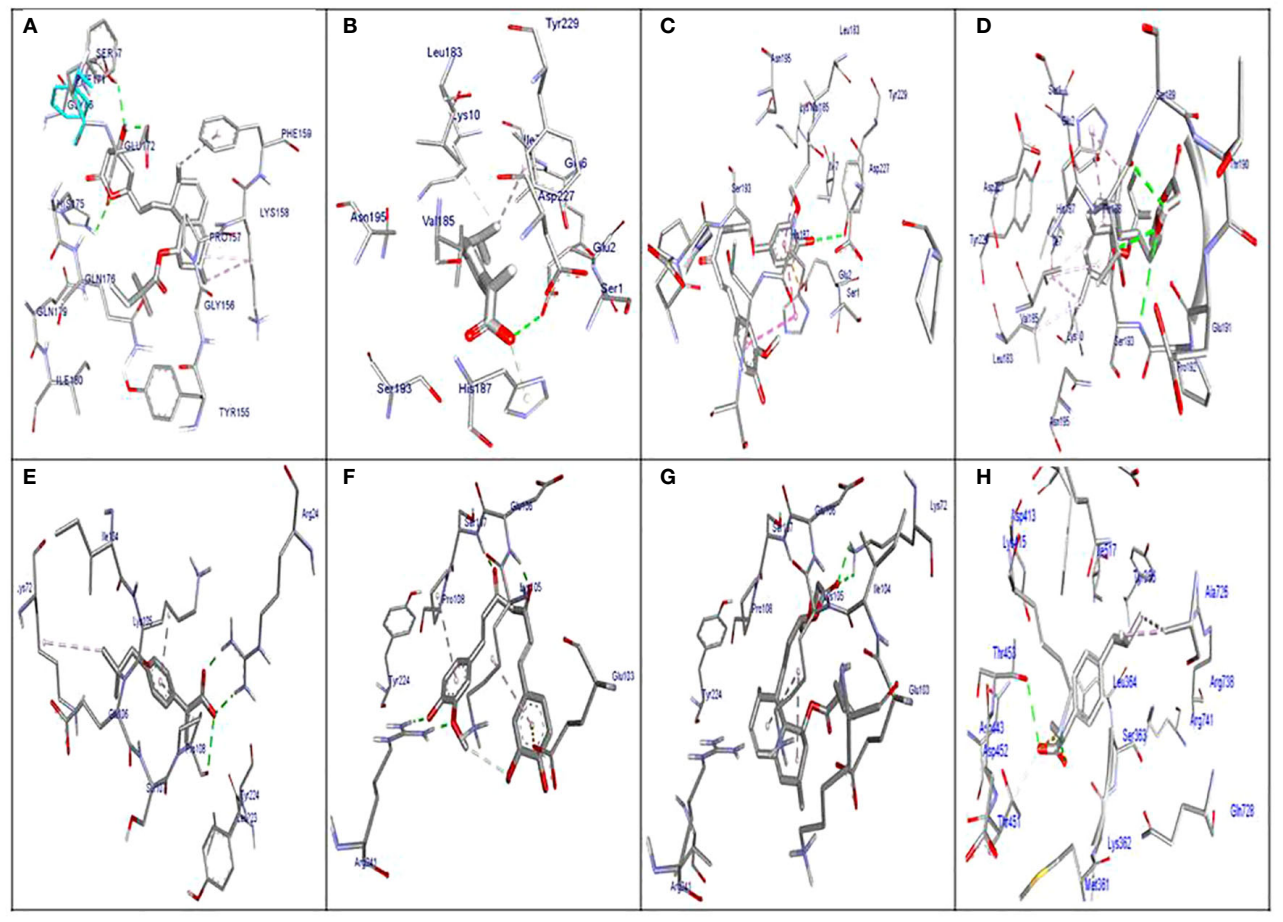
Molecular docking of the investigated compounds with the *tst*, *etb*, *ica*, and enterotoxin virulence genes. **(A)** Visual representation (3D and 2D) of simvastatin showing bond formation and position in the active site of *tst*. **(B–D)** Three-dimensional visual representations of coumarin **(B)**, ibuprofen **(C)**, and simvastatin **(D)** showing bond formation and position in the active site of enterotoxin. **(E, F)** Three-dimensional visual representations of coumarin **(E)** and ibuprofen **(F)** showing bond formation and position in the active site of *etb*. **(G)** Visual representation (3D and 2D) of simvastatin showing bond formation and position in the active site of *etb*. **(H)** Visual representation (3D and 2D) of coumarin showing bond formation and position in the active site of *icaA*.

**Figure 5:**
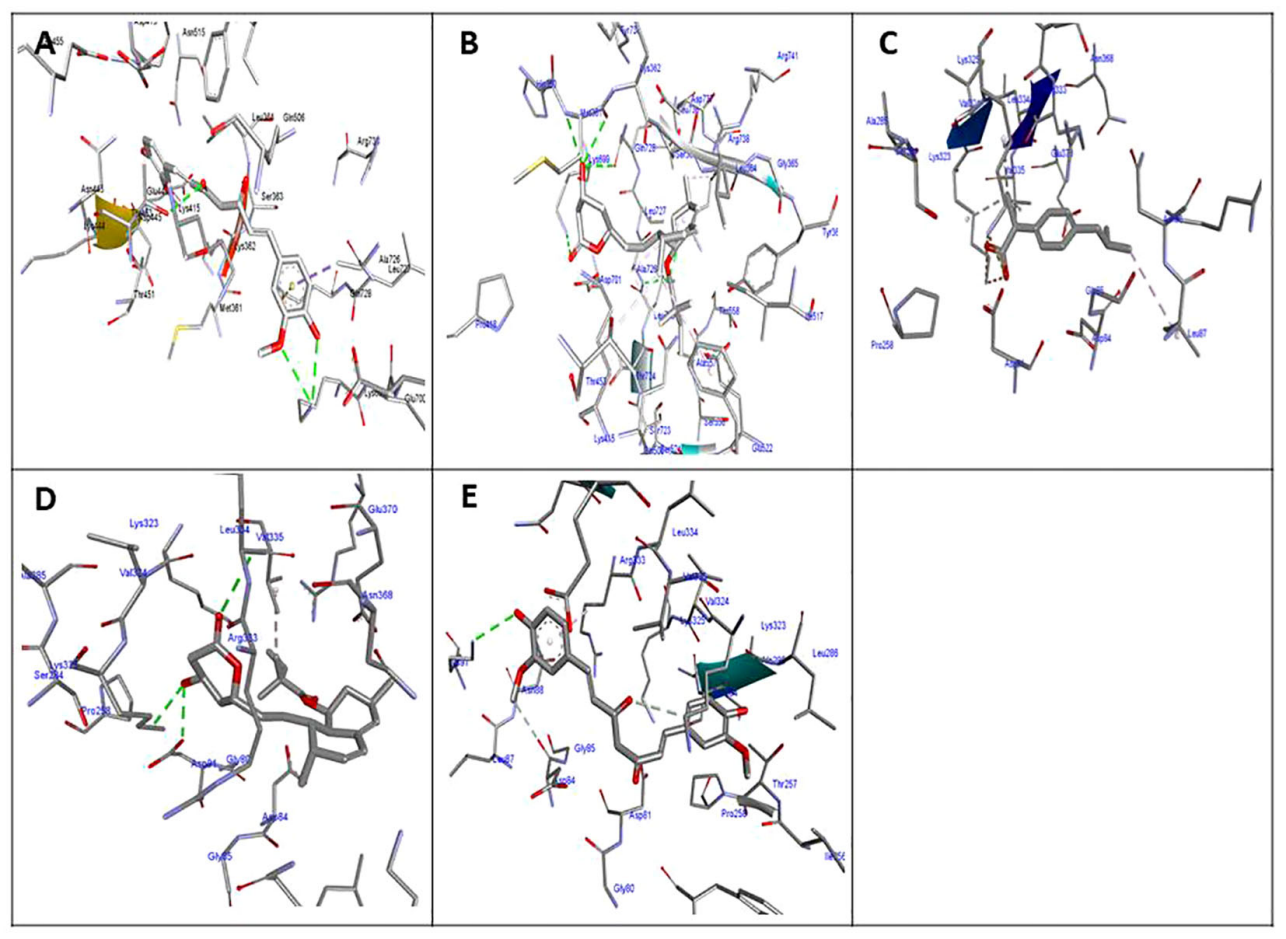
Molecular docking of the investigated compounds with the *icaA* and *hla* virulence genes. **(A, B)** Three-dimensional visual representations of ibuprofen **(A)** and simvastatin **(B)** showing bond formation and position in the active site of *icaA*. **(C–E)** Three-dimensional visual representations of coumarin **(C)**, ibuprofen **(D)**, and simvastatin **(E)** showing bond formation and position in the active site of *hla*.

The molecular docking results indicated that all tested compounds (i.e., coumarin, simvastatin, and ibuprofen) exhibited anti-virulence activities. Ibuprofen displayed the highest affinity for the targets leucocidin ED, exfoliative toxin B, intracellular adhesive toxin A, and hemolysin virulence proteins, with affinity scores of −6.2, −8.3, −15.38, and −12.90, respectively. In contrast, simvastatin showed greater affinity to PBP, TSST, and enterotoxin, with affinity scores of −7.5, −6.5, and −8.3, respectively. Conversely, coumarin exhibited lower affinity to all virulence factors, with affinity scores of −5.4, −6.5, −5, −7.5, −11.84, and −10.84. These docking results are detailed in **Supplementary Table S2** and depicted in **Figures 3**, **4**, and **5**.

### *In vitro* antibacterial and anti-virulence assays for the investigated compounds

Simvastatin demonstrated antibacterial effects at elevated concentrations against two MRSA strains and one VRSA strain (the top three most highly multi-virulent MRSA/VRSA strains), along with the standard *S. aureus* (ATCC 33591), with MICs ranging from 1,250 to 20,000 µg/mL. In addition, this drug exhibited relatively low anti-biofilm activities at sub-MIC values and had no effect on hemolysin production in all investigated isolates. On the other hand, coumarin showed antimicrobial activities, with MICs ranging from 0.763 to 390.6 µg/mL. Moreover, it displayed anti-biofilm and anti-hemolysis activities at sub-MIC values. As for ibuprofen, this drug demonstrated high antimicrobial activities at very low concentrations, with MIC values ranging from 0.24 to 0.10 µg/mL, and exhibited significant anti-biofilm and anti-hemolysis activities at sub-MIC values. Therefore, ibuprofen emerged as the most effective antimicrobial and anti-virulence compound against both MRSA and VRSA when compared with coumarin and simvastatin.

### Assessment of anti-virulence activities through monitoring alterations in the expression of virulence genes

The expression levels of individual virulence genes were standardized using the corresponding bacterial housekeeping gene 16s rRNA. The fold changes in the expression of the virulence genes were assessed in VRSA and MRSA isolates treated with coumarin, simvastatin, and ibuprofen. The findings revealed that ibuprofen exhibited greater anti-virulence efficacy compared with coumarin and simvastatin, as evidenced by the downregulation of all the studied virulence genes upon ibuprofen treatment (**Supplementary Figure S4**).

### Analysis of the effect of ibuprofen on wound healing through *in vivo* and histopathological examination

#### Total aerobic microbial count

Rats treated with vancomycin and the vancomycin/ibuprofen (the most potent anti-virulence drug) combination exhibited a significant decrease in the total microbial count by 95.69% and 99.98%, respectively, compared with the infected, untreated group on day 3. Furthermore, on day 5, the total microbial count decreased in the vancomycin- and vancomycin/ibuprofen-treated groups by 99.99% and 99.96%, respectively. Similarly, on day 14, the treated groups showed a significant decrease in the microbial count by 99.89% and 99.99%, respectively. It is noteworthy that the vancomycin/ibuprofen-treated group displayed a significant decrease in the total microbial count compared with the vancomycin-treated group, as illustrated in **Table 1**.

**Table 1:** Effect of vancomycin and the combination of vancomycin/ibuprofen on total microbial count.

Total microbial count (CFU/g tissue)			
	Day 3	Day 10	Day 14
**Infected, untreated(positive control)**	72 × 10^8^ ± 30,000	10 × 10^6^ ± 284.7	92 × 10^5^ ± 233.3
**Infected, vancomycin-treated group**	31 × 10^7^ ± 29667^a^	12.9 × 10^4^ ± 100^a^	94 × 10^2^ ± 16.7^a^
**Infected, vancomycin/ibuprofen-treated group**	98 × 10^4^ ± 166.7^a,b^	10 × 10^2^ ± 33.3^a,b^	66 ± 1.5^a,b^

Data are expressed as the mean ± SE, one-way ANOVA.

a Significant difference from the infected, untreated group.

b Significant difference from the infected, vancomycin-treated group.

### Effect of vancomycin and the vancomycin/ibuprofen combination on wound healing in mice infected with VRSA

**Figure 6:**
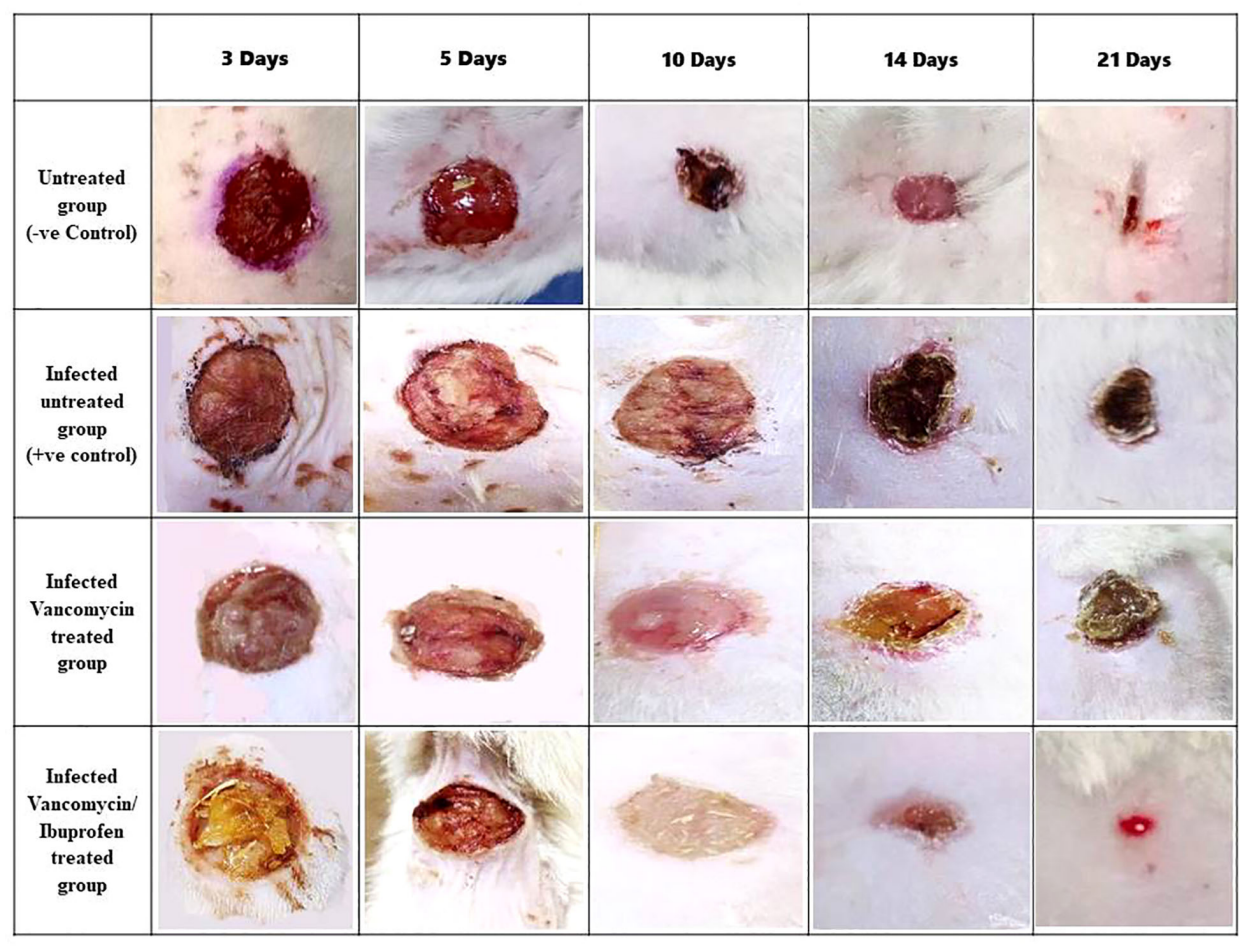
Wound area percentage during the healing process on days 3, 5, 10, 14, and 21. Data are expressed as the mean ± SEM, one-way ANOVA followed by the Tukey–Kramer multiple comparisons test. *p* < 0.05.

There was an insignificant decrease in the wound area percentage in the vancomycin-treated group on days 3, 5, and 10. In contrast, the vancomycin/ibuprofen-treated group exhibited a significant reduction in the wound area percentage on day 3 compared with the infected, untreated group (**Figure 6**). Moreover, there was a noteworthy decrease in the wound area percentage in the vancomycin-treated group by 18.34% and 12.5% on days 14 and 21, respectively, compared with the infected, untreated group. Notably, treatment with the vancomycin/ibuprofen combination significantly reduced the wound area percentage by 23.3% and 17.5% on days 14 and 21, respectively. It is worth mentioning that, on days 14 and 21, the vancomycin/ibuprofen group exhibited a significant decrease in the wound area percentage compared with the vancomycin-treated group (**Figure 6**).

#### Histopathology and healing score

**Figure 7:**
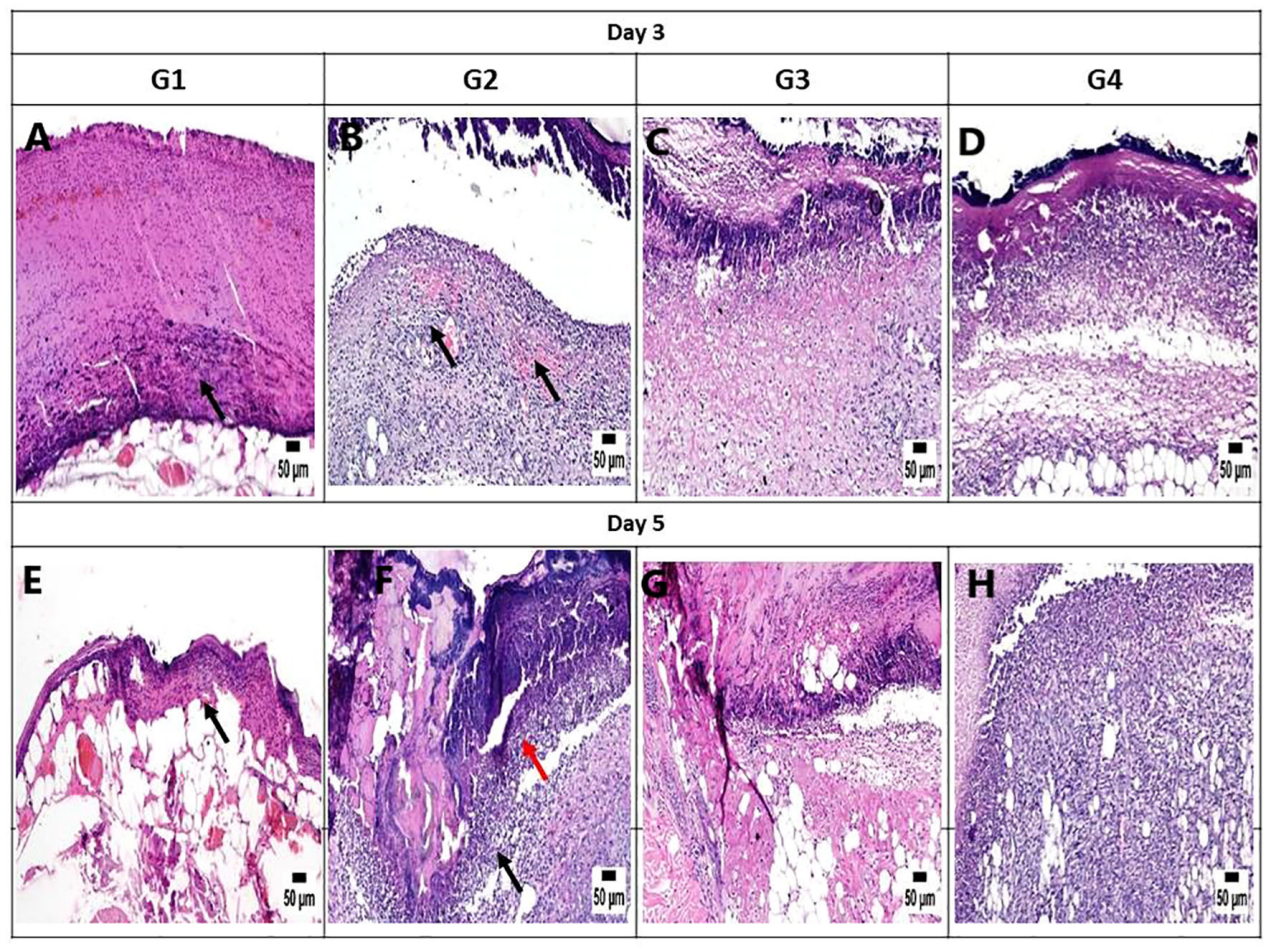
Photomicrograph of the mouse skin wound for the different groups after 3 and 5 days. **(A)** Photomicrograph of the skin in group 1 (G1) at 3 days, higher magnification showing necrotic tissue debris over the wound (*arrow*) (H&E). **(B)** Photomicrograph of the skin in G2 at 3 days showing intense inflammation and hemorrhage in the wound area (*arrow*) (H&E). **(C)** Photomicrograph of the skin in G3 at 3 days, higher magnification showing necrotic tissue in the wound area with mild inflammatory cell infiltration (H&E). **(D)** Photomicrograph of the skin in G4 at 3 days showing necrotic tissue in the wound area with mild inflammatory cell infiltration (H&E). **(E)** Photomicrograph of the skin in G1 at 5 days, higher magnification showing necrotic tissue debris over the wound (*arrow*) (H&E). **(F)** Photomicrograph of the skin in G2 at 5 days showing intense inflammation (*black arrow*) with the presence of bacterial colonies (*red arrow*) (H&E). **(G)** Photomicrograph of the skin in G3 at 5 days, higher magnification showing necrotic tissue in the wound area with mild inflammatory cell infiltration (H&E). **(H)** Photomicrograph of the skin in G4 at 5 days, higher magnification showing granulation tissue formation with inflammatory cell infiltration (H&E).

**Figure 8:**
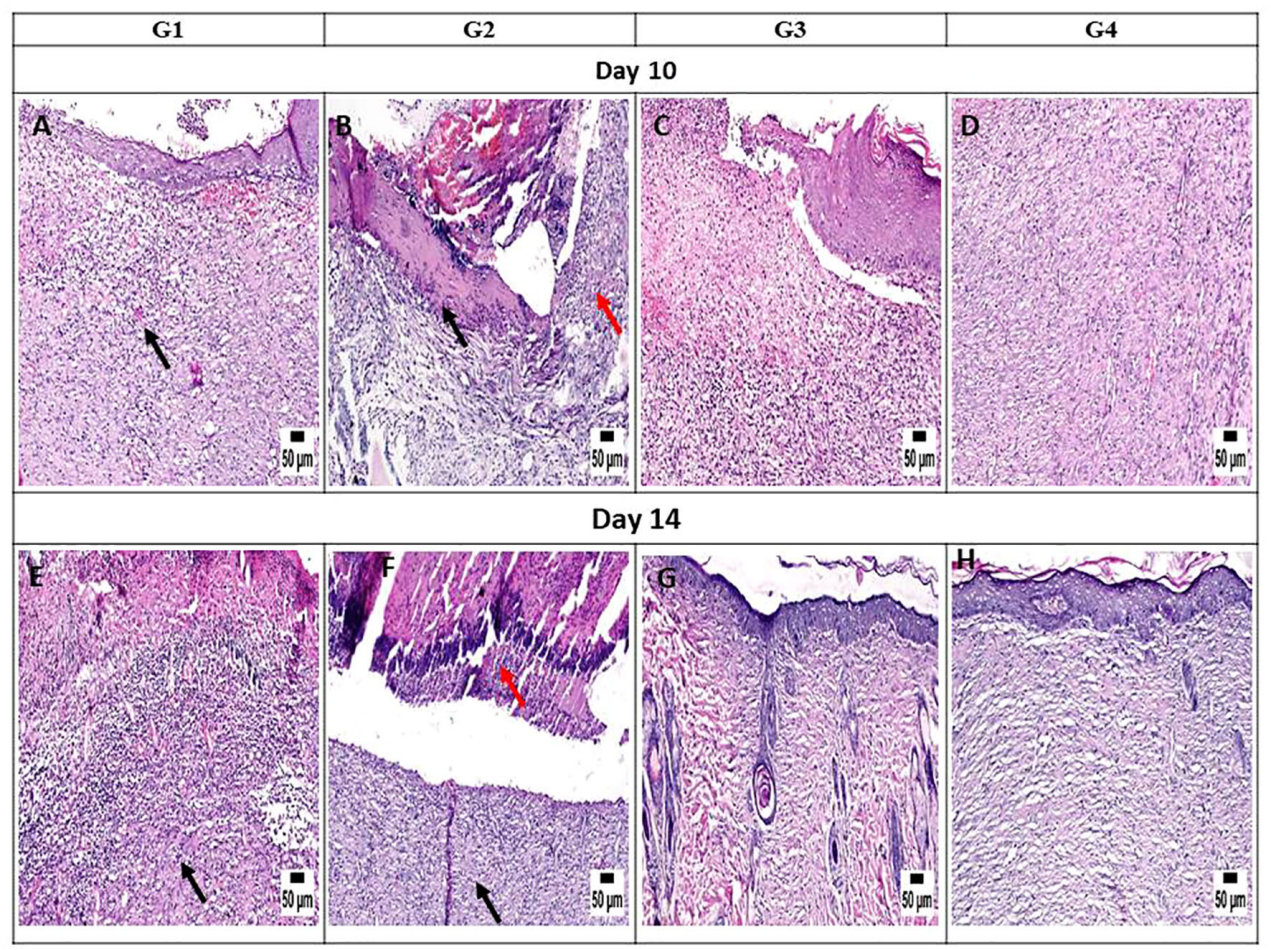
Photomicrograph of the mouse skin wound for the different groups after 10 and 14 days. **(A)** Photomicrograph of the skin in group 1 (G1) at 10 days, higher magnification showing inflamed granulation tissue filling the wound gap (*arrow*) with partial re-epithelization (H&E). **(B)** Photomicrograph of the skin in G2 at 10 days showing intense inflammation (*red arrow*) and necrotic tissue in the wound area (*black arrow*) (H&E). **(C)** Photomicrograph of the skin in G3 at 10 days, higher magnification showing granulation tissue in the wound area, inflammatory cell infiltration, and partial re-epithelization (H&E). **(D)** Photomicrograph of the skin in G4 at 10 days showing organized tissue filling the wound gap with mild inflammatory cell infiltration (H&E). **(E)** Photomicrograph of the skin in G1 at 14 days, higher magnification showing granulation tissue with inflammation (*arrow*) (H&E). **(F)** Photomicrograph of the skin in G2 at 14 days showing intense inflammation (*black arrow*) with the presence of necrotic debris (*red arrow*) (H&E). **(G)** Photomicrograph of the skin in G3 at 14 days, higher magnification showing organized tissue filling the wound area with re-epithelization (H&E). **(H)** Photomicrograph of the skin in G4 at 14 days, higher magnification showing organized tissue filling the wound area with complete re-epithelization (H&E).

On day 3, the histopathological images of the wound area across the different experimental groups exhibited similarities. A thick sero-cellular crust composed of necrotic tissue and inflammatory exudate covered the wound gap. Inflamed granulation tissue and hemorrhagic exudate were observed in some cases. The degree of inflammation appeared to decrease in G3 and G4 (**Figures 7A-D**). By day 5, G1 displayed a thick crust comprising necrotic tissue debris and infiltrating inflammatory cells, hindering re-epithelialization and granulation tissue formation. G2 exhibited intense infiltration of inflammatory cells at the wound site along with abundant necrotic tissue and bacterial clusters. Despite the presence of necrotic tissue in G3, inflammation was confined to the upper layer of the wound. In G4, granulation tissue formation was evident, accompanied by numerous capillaries despite the pronounced inflammatory response (**Figures 7E-H**). On day 10, G1 presented with granulation tissue filling the wound gap along with significant inflammatory cell infiltration. Partial re-epithelialization was observed at the wound edges. G2 displayed a marked inflammatory cell infiltration and delayed re-epithelialization, with increased amounts of necrotic tissue in the wound gap. G3 exhibited partial re-epithelialization at the wound edges with granulation tissue filling the wound area, accompanied by moderate inflammatory cell infiltration. G4 showcased collagen-rich organized tissue filling the wound area, along with numerous newly formed blood vessels. Partial re-epithelialization was also noted, accompanied by mild inflammatory cell infiltration (**Figures 8A-D**).

**Figure 9:**
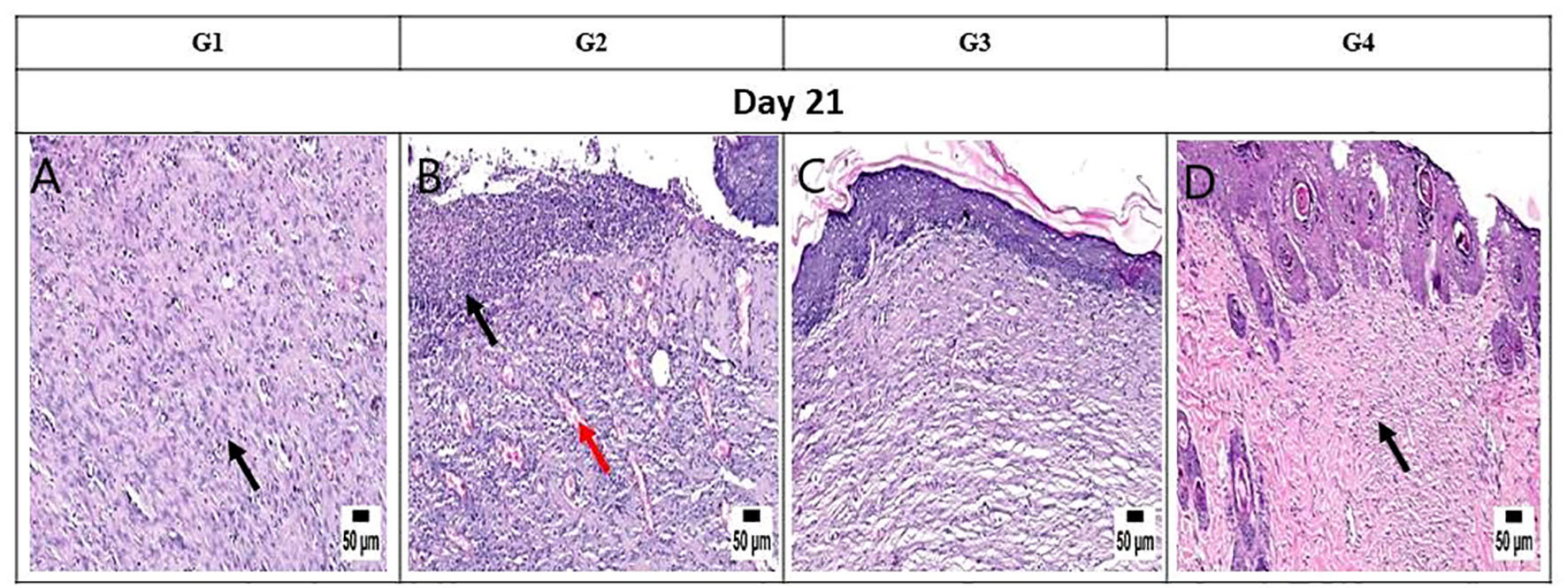
Photomicrograph of the mouse skin wound for the different groups after 21 days. **(A)** Photomicrograph of the skin in group 1 (G1) at 21 days, higher magnification showing granulation tissue filling the wound gap (*arrow*) with inflammatory cell infiltration (H&E). **(B)** Photomicrograph of the skin in G2 at 21 days, higher magnification showing intense inflammation (*arrow*) and numerous dilated vessels in the wound area (*red arrow*) (H&E). **(C)** Photomicrograph of the skin in G3 at 21 days, higher magnification showing complete re-epithelization and organized tissue filling the wound area (H&E). **(D)** Photomicrograph of the skin in G4 at 21 days, higher magnification showing collagen-rich organized tissue in the wound gap with complete re-epithelization (H&E).

On day 14, notable observations included delayed re-epithelialization in G1, with granulation tissue present and a marked infiltration of inflammatory cells filling the wound area. G2 displayed intense inflammation within the formed granulation tissue, coupled with delayed re-epithelialization and increased necrotic tissue debris. G3 exhibited significant improvement, with complete re-epithelialization and organized tissue filling the wound area, accompanied by mild inflammation. Similarly, G4 showed enhanced healing, with a wound surface fully covered by newly formed epithelium. Collagen-rich organized tissue filled the wound gap, showing minimal inflammation and numerous blood vessels (**Figures 8E-H**). Interestingly, by day 21, G1 demonstrated a granulation tissue core filling the wound gap, with delayed re-epithelialization and infiltration of inflammatory cells. The formed granulation tissue exhibited numerous dilated blood vessels and a marked inflammatory cell infiltration. G2 showed only partial re-epithelialization, while G3 displayed complete re-epithelialization, with the wound surface covered by newly formed epithelium. Organized tissue filled the wound gap, featuring numerous capillaries and minimal infiltration of inflammatory cells. G4 exhibited significant wound contraction and re-epithelialization, with the wound gap filled with collagen-rich organized tissue showing minimal inflammation. The wound surface was covered by hyperplastic newly formed epithelium (**Figures 9A-D**).

It is worth noting that the healing scores at different time intervals consistently showed superior healing criteria in G4, closely followed by G3. These groups demonstrated a notable reduction in inflammation during the initial stages of sacrifice compared with the untreated groups (G1 and G2). Later on, G3 and G4 exhibited improved re-epithelialization and formation of organized tissue. In contrast, both untreated groups showed increased inflammation toward the end of the experiment.

## Discussion

Serious complications from skin, soft tissue, bone, and lung infections, which contribute to increased morbidity and mortality globally, are often caused by MRSA strains. Unfortunately, the situation has worsened due to the widespread emergence of VRSA strains. Despite extensive efforts over the years to develop new antimicrobial drugs to combat MRSA and VRSA, treatment failures and poor prognoses remain common. Therefore, we aimed to address this crisis by investigating the anti-virulence activities of certain natural (coumarin) and synthetic (simvastatin and ibuprofen) compounds. Notably, the pathogenicity of *S. aureus* is attributed to its production of numerous virulence factors, including secreted toxin proteins (e.g., hemolysin), extracellular polysaccharides (e.g., intercellular adhesion), and other elements that facilitate infection processes (Karmakar et al., 2016).

Resistance to methicillin, macrolides, aminoglycosides, and lincosamides, as well as combinations of these antimicrobial drugs, has been frequently reported (Bendary et al., 2016a). *S. aureus* has developed resistance not only to methicillin but also to many commonly used antibiotics. As new antimicrobial drugs are introduced, it is likely that this highly adaptable pathogen will develop resistance to them as well. In this study, all of the detected MRSA strains exhibited MDR patterns, and the antimicrobial resistance patterns of the VRSA strains were particularly alarming. The widespread occurrence of the MDR strains in this study may be attributed to the overuse or misuse of various broad-spectrum antibiotics, particularly β-lactams such as penicillins and cephalosporins. This indiscriminate use leads to the development of resistance and an increased risk of MRSA infection, corroborating findings from a previous study conducted in Iraq by Algammal et al. (2020).

Notably, infections caused by MDR–MRSA strains are often treated empirically with antibiotics that are ineffective against MRSA, which can exacerbate the antibiotic resistance (Romeo, 2008). Unfortunately, several studies have reported an increase in the prevalence of VRSA strains (Bendary et al., 2016b; Abd El-Aziz et al., 2018). Vancomycin is generally the drug of choice for the treatment of serious MRSA and MSSA infections. However, the irrational use of antibiotics has led to the emergence of VRSA strains (Khanam et al., 2016). Consequently, the MRSA and VRSA infections caused by MDR strains pose severe global health crises, making the development of novel antimicrobial drugs urgently necessary. To date, the development of new drugs with clear antibacterial mechanisms for MDR pathogens remains a significant challenge (Zhang et al., 2021).

The ability of staphylococci to produce biofilms is recognized as an important virulence trait (Vancraeynest et al., 2004). In this study, 48.1% of the MRSA isolates were confirmed to be biofilm producers using quantitative MTP methods, with genetic confirmation through the detection of the *icaD* and *icaA* genes. Another study reported a higher prevalence of biofilm-producing MRSA and VRSA strains (Saber et al., 2022). With regard to the hemolytic activity, 85.19% of the tested MRSA isolates in this study produced hemolysins, indicating significant hemolytic activity. In addition, most of the MRSA strains possessed hemolysin toxin-producing genes (Dunyach-Remy et al., 2016). Variations in biofilm and hemolysin production may be attributed to factors such as the geographical origin, the type of specimen, and genetic mutations of the *S. aureus* isolate. Environmental conditions, including the surface type (rough or smooth), surface charge, porosity, and growth medium, can also influence biofilm formation. These factors have been discussed in previous studies (Abdelraheem et al., 2021; Silva et al., 2021).

On the other hand, infections with MRSA strains are problematic due to the widespread presence of various clones carrying multiple antimicrobial resistance genes. The degree of microbial pathogenesis depends on the presence of virulence genes, and the absence of these genes is typically associated with non-pathogenic strains (Elsayed et al., 2022; Eibach et al., 2017). Unfortunately, the virulence profiles of the isolates investigated in this study were alarming, with all exhibiting multi-virulent profiles. The high frequency of certain virulence genes may indicate the emergence of multi-virulent isolates (Hoseini Alfatemi et al., 2014). Consequently, a new therapeutic strategy is needed to interfere with pathogen virulence arrays, preventing microbial pathogenesis without killing the microbes and, thus, applying less evolutionary pressure on resistance development. The discovery of new anti-virulence compounds is crucial for improving treatment success and prognosis in infectious diseases.

Molecular docking is a crucial tool for the development of new drugs. This technique models the interaction between a promising drug and its receptor at the atomic level, allowing researchers to understand fundamental biochemical processes and to assess the binding affinity with the target protein (McConkey et al., 2002). In this study, molecular docking analysis predicted the affinity scores of coumarin, simvastatin, and ibuprofen to specific virulence protein targets *in vitro*. The results demonstrated that all of these compounds have anti-virulence activities, with ibuprofen showing the highest affinity scores for most of the investigated virulence proteins. These anti-virulence compounds present a promising alternative therapy for effectively managing and treating infections with resistant MRSA strains. The molecular docking results were corroborated by phenotypic evaluations of the anti-biofilm and anti-hemolytic activities and were further confirmed genotypically using real-time PCR. All investigated virulence genes were downregulated upon treatment with these compounds, showing fold changes of less than 1, with the lowest levels of downregulation observed post-treatment with ibuprofen. These findings align with other studies suggesting that ibuprofen is a good candidate for repurposing as an antimicrobial agent (Obad et al., 2015; Oliveira et al., 2019). In addition, biofilm formation increased gradually with decreasing drug concentration, supporting previous research indicating that ibuprofen may be effective as an anti-biofilm agent and could be a viable alternative for the treatment of active and mature biofilms produced by *S. aureus* (Oliveira et al., 2019; Paes-Leme and da Silva, 2021).

Undeniably, wound infections caused by MRSA and VRSA strains are a significant global problem. Topical antibacterial and anti-inflammatory formulations can offer improved benefits for treating infected wounds. Previous studies have shown that local administration of ibuprofen provides a pronounced anti-inflammatory effect and reduces pain with smaller doses compared with oral administration. Consequently, the risk of side effects from local application is very low, making it much safer than oral ibuprofen (Sibbald et al., 2006). In addition, the potential antibacterial effect of ibuprofen has been observed in isolated bacterial strains (Al-Janabi, 2010).

Infected wound healing involves complex mechanisms, including the proliferative phase, hemostasis/inflammatory phase, and scar tissue remodeling, which cannot be fully replicated *in vitro* (Abbasi et al., 2022). Therefore, this *in vivo* study was designed to assess the combined effect of local ibuprofen and vancomycin on the treatment of wound infections and the improvement of wound healing. The study demonstrated that treating wound infections caused by VRSA strains with a combination of ibuprofen and vancomycin exhibited superior antibacterial activity compared with vancomycin alone. The combination effectively reduced the total microbial count of the wound over various time intervals (days 3, 5, 10, 14, and 21). In addition, this combination significantly decreased the wound area percentage, reflecting better wound healing, particularly on days 14 and 21. Notably, these results support the concept that the combination of ibuprofen/vancomycin may represent a promising new therapy for the treatment of wound infections and improvement of wound healing due to its superior antibacterial capacity and anti-inflammatory activity. Moreover, the mouse protection assay, which has been used in several studies, confirmed the antimicrobial and anti-virulence activities of some new drugs (Bourne et al., 1999; Hegazy et al., 2020).

In conclusion, MRSA and VRSA strains were frequently identified in several cases discussed in this report. This study confirmed resistance to last-resort anti-staphylococcal drugs such as linezolid and pristinamycin. Fortunately, the repurposing of simvastatin and ibuprofen (FDA-approved drugs) as new anti-virulence agents has shown promising potential without the need for extensive human trials. Interestingly, combining ibuprofen with other anti-staphylococcal drugs has been proven effective in treating MRSA and VRSA infections. These promising results, given the lack of novel antimicrobial drugs and the slow pace of new drug development, can be applied in clinical settings. Fortunately, no further safety evaluations or pharmacokinetic studies are required for these compounds to be used in different countries.

## Data Availability

The original contributions presented in the study are included in the article/**Supplementary Material**. Further inquiries can be directed to the corresponding author.
