# Supplementary material for: Proven anti-virulence therapies in combating methicillin- and vancomycin-resistant Staphylococcus aureus infections
Source: Front Cell Infect Microbiol. 2024 Aug 26;14:1403219. doi: 10.3389/fcimb.2024.1403219 (PMC11381379; doi:10.3389/fcimb.2024.1403219)
Supplement: Supplementary file 1 [file DataSheet1.docx]

**Table S1: Oligonucleotide primers and annealing temperature for the investigated virulence genes**

| **Reference** | **Annealing**  **Temperature** | **Length of amplified product** | **Primer sequence**  **(5'-3')** | **Gene** |
| --- | --- | --- | --- | --- |
| **Mehrotra *et al*., 2000** | 57˚C  40 sec | 102 bp | GGTTATCAATGTGCGGGTGG | ***Sea*** |
|  |  |  | CGGCACTTTTTTCTCTTCGG |  |
| **Mehrotra *et al*., 2000** | 57˚C  40 sec | 164 bp | GTATGGTGGTGTAACTGAGC | ***Seb*** |
|  |  |  | CCAAATAGTGACGAGTTAGG |  |
| **Mehrotra *et al*., 2000** | 57˚C  40 sec | 451 bp | AGATGAAGTAGTTGATGTGTATGG | ***Sec*** |
|  |  |  | CACACTTTTAGAATCAACCG |  |
| **Mehrotra *et al*., 2000** | 57˚C  40 sec | 278 bp | CCAATAATAGGAGAAAATAAAAG | ***Sed*** |
|  |  |  | ATTGGTATTTTTTTTCGTTC |  |
| **Mehrotra *et al*., 2000** | 57˚C  40 sec | 209 bp | AGGTTTTTTCACAGGTCATCC | ***See*** |
|  |  |  | CTTTTTTTTCTTCGGTCAATC |  |
| **Mehrotra *et al*., 2000** | 50˚C  30 sec | 226 bp | ACAAGCAAAAGAATACAGCG | ***Etb*** |
|  |  |  | GTTTTTGGCTGCTTCTCTTG |  |
| **Mehrotra *et al*., 2000** | 50˚C  40 sec. | 326 bp | ACCCCTGTTCCCTTATCATC | ***tst*** |
|  |  |  | TTTTCAGTATTTGTAACGCC |  |
| **Ciftci *et al*. 2009** | 49˚C  1 min. | 1315 bp | CCT AAC TAA CGA AAG GTA G | ***icaA*** |
|  |  |  | AAG ATA TAG CGA TAA GTG C |  |
| **Ciftci *et al*. 2009** | 49˚C  40 sec. | 381 bp | AAA CGTAAG AGA GGT GG | ***icaD*** |
|  |  |  | GGC AAT ATG ATC AAGATA |  |
| **Fei *et al*., 2011** | 53˚C  40 sec. | 704 bp | GAAGTCTGGTGAAAACCCTGA | ***Hla*** |
|  |  |  | TGAATCCTGTCGCTAATGCC |  |
| **Fei *et al*., 2011** | 53˚C  40 sec. | 496 bp | CAATAGTGCCAAAGCCGAAT | ***hlb*** |
|  |  |  | TCCAGCACCACAACGAGAAT |  |
| [**Bronner**](https://pubmed.ncbi.nlm.nih.gov/?term=Bronner+S&cauthor_id=10966411) ***et al*., 2000** | 56˚C  40 sec. | 516 bp | TAGGCAAATCATCAGTTGCTTCAT | ***lukED*** |
|  |  |  | GTAGTTCTGTAACTTTCTTGTTT |  |
| **Kumar *et al*., 2009** | 55˚C  40 sec. | 937 bp | GCCAATCCGTTATTAGAAAATGC | ***hlg*** |
|  |  |  | CCATAGACGTAGCAACGGAT |  |
| **Levinger *et al*., 2012** | 58˚C  1 min. | 55˚C  40 sec | ACGTCGTTAACAGAAACCAAGCA | ***Pbp2*** |
|  |  |  | TGCGGTTGGTCATGAATTAGG |  |
| **Mason *et al.,* 2001** | 55˚C  1 min. | 55˚C  40 sec. | CCTATAAGACTGGGATAACTTCGGG | ***16S rRNA*** |
|  |  |  | CTTTGAGTTTCAACCTTGCGGTCG |  |

**Table S2: the scores of molecular docking and basic chemical properties of the studied ligands**

| **The Drugs** | **Docking scores** | **Binding type** | **Distance** |
| --- | --- | --- | --- |
| **Coumarin** | -10.84 | Salt Bridge  Attractive Charge  Attractive Charge  Attractive Charge  Alkyl  Alkyl  Alkyl  Alkyl | 2.90616  4.83465  3.7202  4.51977  4.80656  4.07346  4.4509  4.7927 |
| **Ibuprofen** | -12.90 | Conventional Hydrogen Bond  Carbon Hydrogen Bond  Carbon Hydrogen Bond  Pi-Alkyl | 3.06298  3.51001  3.23772  3.9989 |
| **Simvastatin** | -11.90 | Conventional Hydrogen Bond  Conventional Hydrogen Bond  Conventional Hydrogen Bond  Alkyl | 2.84413  3.11873  2.7198  4.0206 |


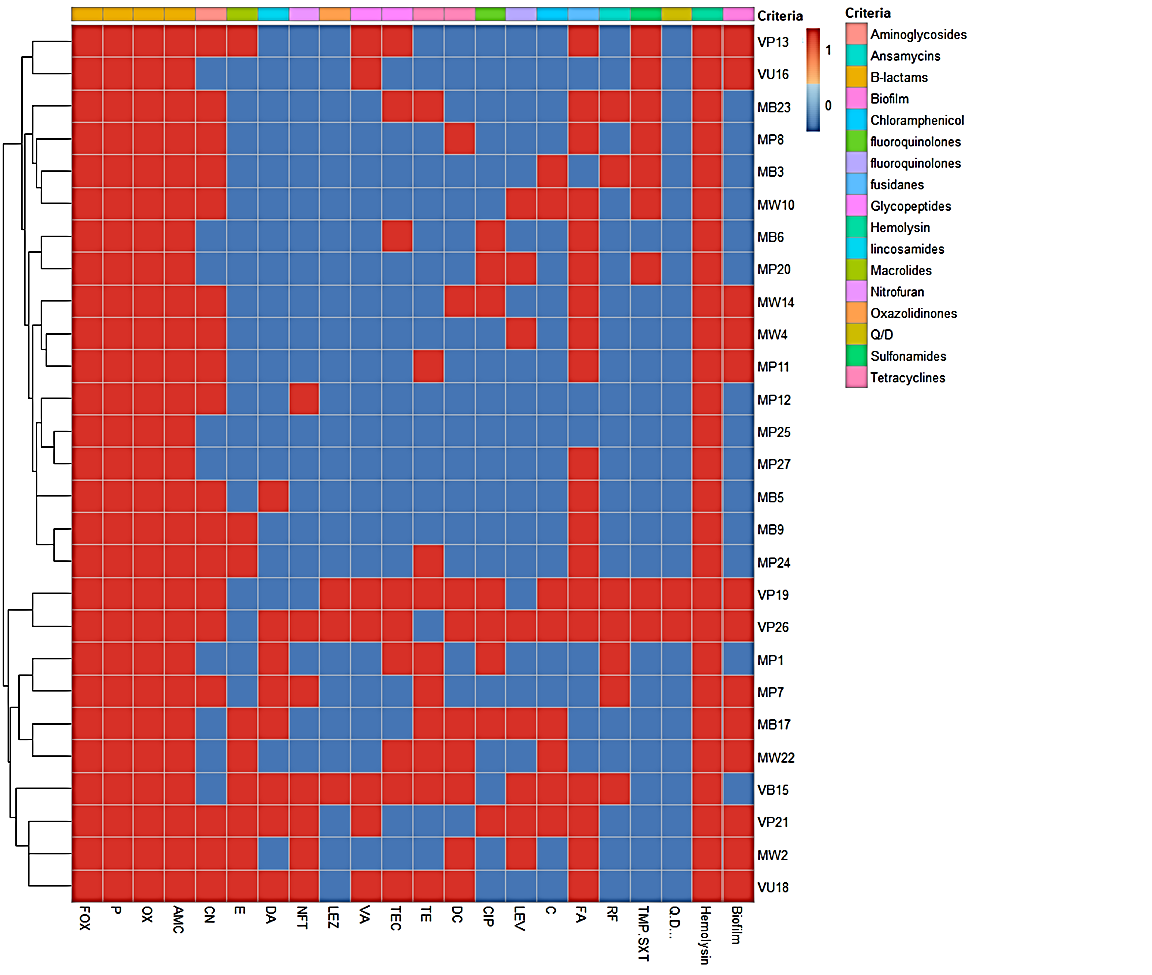


**Fig. S1: Heat map represent the distribution of MRSA strains based on antimicrobial resistance profiles, biofilm and hemolysin production**

The isolates code: M and V represent MRSA and VRSA strains respectively. However, the second letter refer to the type of samples from which the isolate was recovered (P; pus, B; blood, U; urine, W; wound swab), and the numerical values refer to the order of recovery. The red and blue colors codes refer to the presence or absence of resistance to each antimicrobial, biofilm, and hemolysin production respectively. Fox: Cefoxitin, P: Benzylpenicillin, OX: Oxacillin, AMC: Amoxicillin/clavulanic acid, CN: Gentamicin, E: Erythromycin, DA: Clindamycin, NFT: Nitrofurantoin, LEZ: Linezolid, VA: Vancomycin, TEC: Teicoplanin, TE: Tetracycline, DC: Doxycycline, CIP: Ciprofloxacin, LEV: Levofloxacin, C: Chloramphenicol, FA: Fusidic Acid, RF: Rifampicin, TMP/SXT: Cotrimoxazole, QD: Pristinomycin (Quinupristin/Dalfopristin).

**
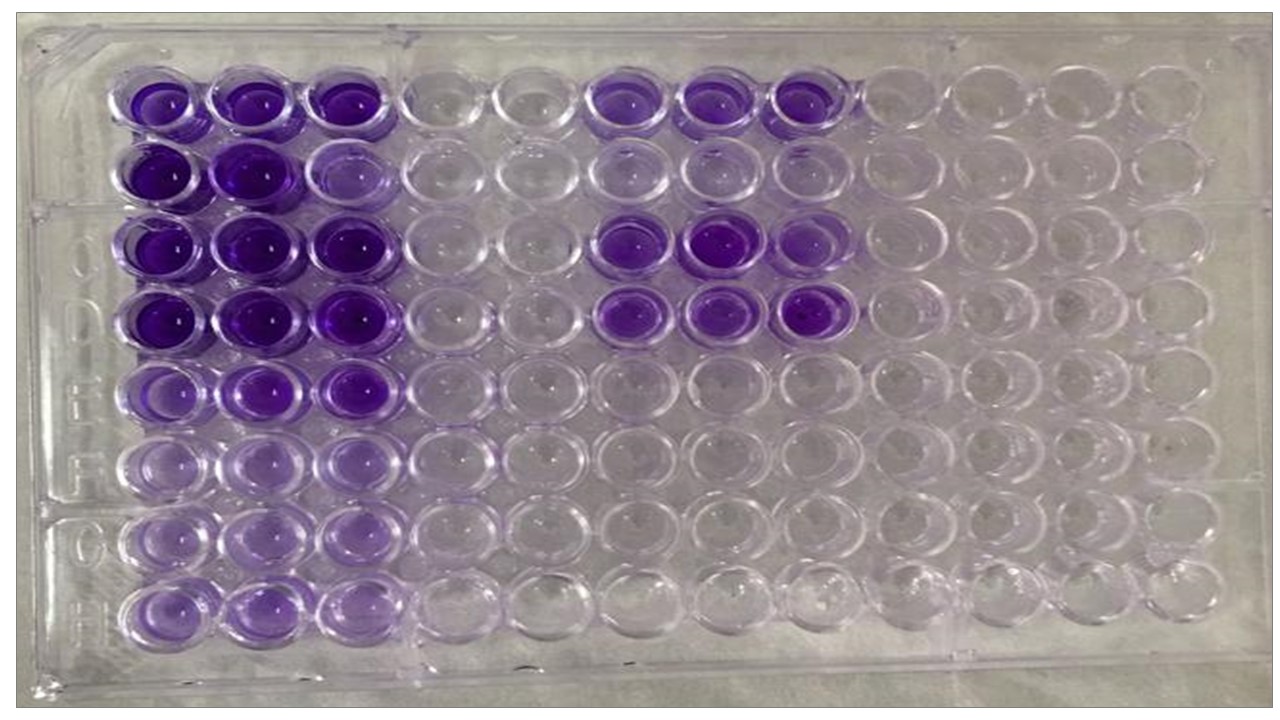
**

**Fig. S2 : Quantification of Biofilm Production by MRSA Strains Using Microtitre Plate Assay with Crystal Violet Staining**

**
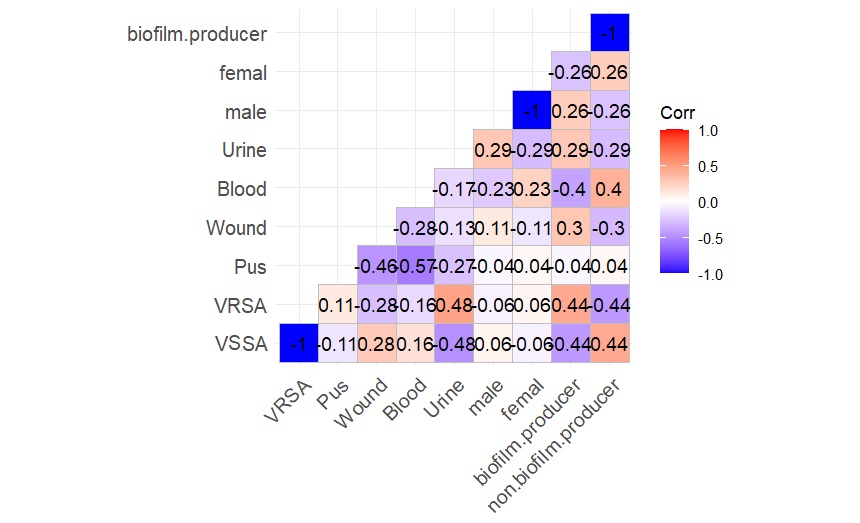
**

**Fig. S3 : Heatmap displaying the correlation coefficient (r) values between biofilm production and clinical samples, as well as different strain types**

The color key is provided on the right side. Stronger positive or negative correlations are represented by darker red and blue colors, respectively.


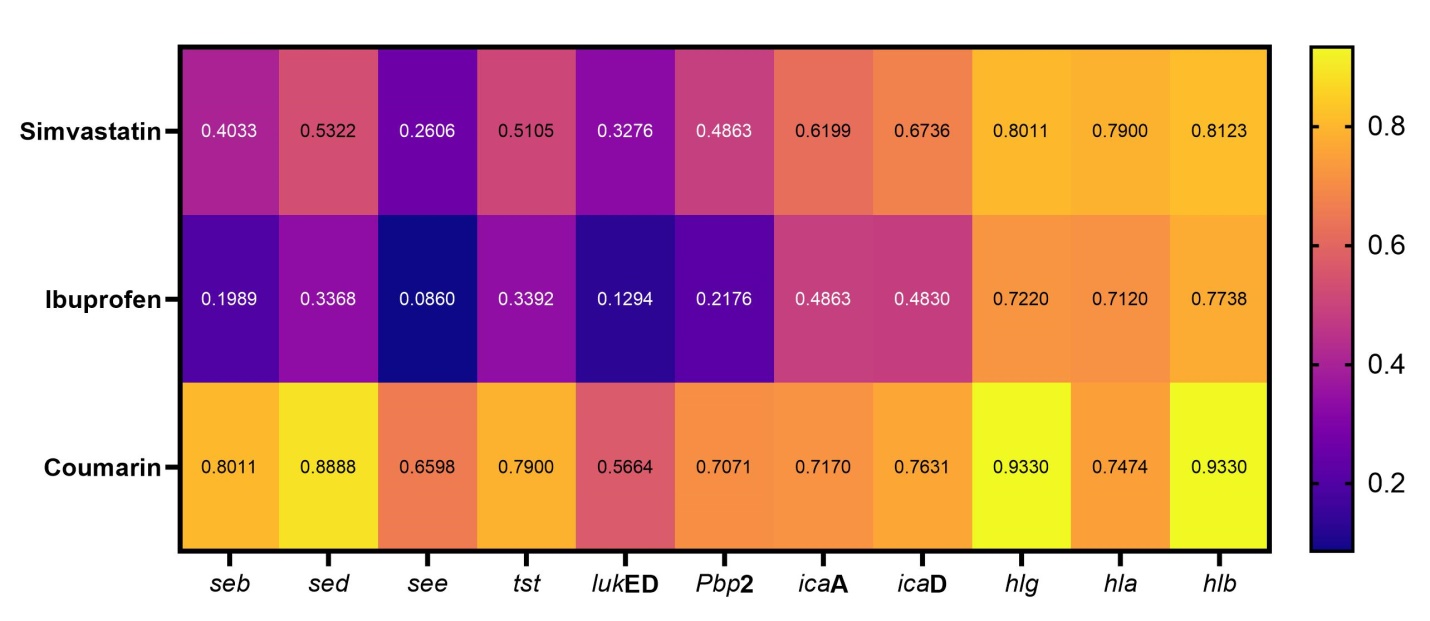


**Fig. S4: Heat map represent the fold change in the virulence gene expression post treatment with the investigated compounds.**

The deep blue and yellow colors refer to the lowest (0) and highest (10) fold change values. *Sea, Seb, Sec, Sed* and *See:* Staphylococcal Enterotoxins A,B,C,D,E genes, *hla, hlb, hlg:* Hemolysins A,B,G genes, *icaA* and *icaD:* Intracellular adhesive toxin A and D, *tst:* Toxic shock syndrome toxin gene, *etb:* exfoliative B gene, *LukED:*  Leukocidin gene

**References**

Mehrotra, M., Wang, G., Johnson, W.M. (2000). Multiplex PCR for Detection of Genes for *Staphylococcus aureus* Enterotoxins., Exfoliative Toxins., Toxic Shock Syndrome Toxin 1., and Methicillin Resistance. J. Clin. Microbiol.. 38: 3.

Ciftci, A., Findik, A., Onuk, E. E., & Savasan, S. (2009). Detection of methicillin resistance and slime factor production of *Staphylococcus aureus* in bovine mastitis. *Brazilian journal of microbiology : [publication of the Brazilian Society for Microbiology]*, *40*(2), 254–261. https://doi.org/10.1590/S1517-83822009000200009

Fei, W., Hongjun, Y., Hong-bin, H., Changfa, W., Yundong, G., Qifeng, Z., Wiaohong, W., & Yanjun, Z. (2011). Study on the hemolysin phenotype and the genotype distribution of *Staphylococcus aureus* causing bovine mastitis in Shandong dairy farms. International Journal of Applied Research in Veterinary Medicine, 9(4), pp. 416-421.

Bronner, S., Stoessel, P., Gravet, A., Monteil, H., & Prévost, G. (2000). Variable expressions of *Staphylococcus aureus* bicomponent leucotoxins semiquantified by competitive reverse transcription-PCR. *Applied and environmental microbiology*, *66*(9), 3931–3938. <https://doi.org/10.1128/AEM.66.9.3931-3938.2000>

Kumar, J.D., Negi, Y.K., Gaur, A., Khanna, D. (2009) Detection of virulence genes in *Staphylococcus aureus* isolated from paper currency.  Int. J. Infect. 13: e450-e455.

Levinger, O., Bikels-Goshen, T., Landau, E., Fichman, M., Shapira, R. (2012). Epigallocatechin Gallate Induces Upregulation of the Two-Component VraSR System by Evoking a Cell Wall Stress Response in *Staphylococcus aureus.* Appl. Environ. Microbiol. 78 (22):7954–7959

Mason, W. J., Blevins, J. S., Beenken, K., Wibowo, N., Ojha, N., & Smeltzer, M. S. (2001). Multiplex PCR protocol for the diagnosis of staphylococcal infection. *Journal of clinical microbiology*, *39*(9), 3332–3338. https://doi.org/10.1128/JCM.39.9.3332-3338.2001
